# Metabolic and lifestyle factors accelerate disease onset and alter gut microbiome in inflammatory non-communicable diseases

**DOI:** 10.1186/s12916-024-03709-0

**Published:** 2024-10-24

**Authors:** Nathalie Rohmann, Theresa Geese, Samantha Nestel, Kristina Schlicht, Corinna Geisler, Kathrin Türk, Fynn Brix, Julia Jensen-Kroll, Tobias Demetrowitsch, Corinna Bang, Andre Franke, Wolfgang Lieb, Dominik M. Schulte, Karin Schwarz, Anne-Kathrin Ruß, Arunabh Sharma, Stefan Schreiber, Astrid Dempfle, Matthias Laudes

**Affiliations:** 1 Institute of Diabetes and Clinical Metabolic Research, University Medical Center Schleswig-Holstein, Düsternbrooker Weg 17, Kiel, 24105 Germany; 2 Institute for Medical Informatics and Statistics, University Medical Center Schleswig-Holstein, Kiel, Germany; 3 Division of Food Technology, Institute of Human Nutrition and Food Sciences, Kiel University, Kiel, Germany; 4 Institute of Clinical Molecular Biology, Kiel University, Kiel, Germany; 5 Institute of Epidemiology, Kiel University, Kiel, Germany; 6 Division of Endocrinology, Diabetes and Clinical Nutrition, Department of Internal Medicine I, University Medical Center Schleswig-Holstein, Kiel, Germany; 7 Department of Internal Medicine I, University Medical Center Schleswig-Holstein, Kiel, Germany

**Keywords:** Diabetes, Inflammatory bowel disease, Biomedical and lifestyle factors, Diet scores, Age-at-disease-onset, Gut microbiome composition

## Abstract

**Background:**

Biomedical and lifestyle factors in Western populations have significantly shifted in recent decades, influencing public health and contributing to the increasing prevalence of non-communicable diseases (NCDs) that share inflammation as common pathology.

**Methods:**

We investigated the relationship between these factors and 11 NCDs in the cross-sectional FoCus cohort (*n* = 1220), using logistic regression models. Associations with age-at-disease-onset were specifically analyzed for type 2 diabetes (T2D, low-grade chronic inflammation) and inflammatory bowel disease (IBD, high-grade chronic inflammation) in disease-specific cohorts (FoCus-T2D, *n* = 514; IBD-KC, *n* = 1110). Important factors for disease risk were identified using Cox-PH-regression models and time-to-event analysis. We further explored the interaction between identified risk factors and gut microbiome composition using linear models.

**Results:**

Lifestyle factors were clearly linked to disease phenotypes, particularly in T2D and IBD. Still, some factors affected only the age-at-onset, but not disease prevalence. High-quality nutrition significantly delayed onset for both IBD and T2D (IBD: HR = 0.81 [0.66; 0.98]; T2D: HR = 0.45 [0.28; 0.72]). Smoking accelerated T2D onset (HR = 1.82 [1.25; 2.65]) but delayed onset in ulcerative colitis (UC: HR = 0.47 [0.28; 0.79]). Higher microbiota diversity delayed IBD onset (Shannon: HR = 0.58 [0.49; 0.71]) but had no effect on T2D. The abundance of specific microbial genera was strongly associated with various biomedical and lifestyle factors in T2D and IBD. In unaffected controls, these effects were smaller or reversed, potentially indicating a greater susceptibility of the gut microbiome to negative influences in T2D and IBD.

**Conclusions:**

The dual insights into age-at-disease-onset and gut microbiota composition in disease emphasize the role of certain biomedical and lifestyle factors, e.g., nutrition quality, in disease prevention and management. Understanding these relationships provides a foundation for developing targeted strategies to mitigate the impact of metabolic and inflammatory diseases through lifestyle modifications and gut health management.

**Supplementary Information:**

The online version contains supplementary material available at 10.1186/s12916-024-03709-0.

## Background

Biomedical and lifestyle factors, combining anthropometric, lifestyle, metabolic and disease-specific factors, of the western population have drastically changed during the last decades. Main alterations can be observed in, e.g., (1.) nutrition: a transition in dietary patterns marked by increased consumption of processed food, often high in sugar and a growing fast food culture leading to changes in food choices and portion sizes [1], (2.) physical activity: technological advancements and changes in work environments leading to a more sedentary lifestyle and a decline in overall physical activity [2], (3.) anthropometric factors: the obesity rate increased significantly and an association with sleeping patterns is observed [3], (4.) environmental factors: Urbanization and associated lifestyle choices and increased stress levels potentially leading to a decline in mental health [4]. Simultaneously, the medical system is faced with the consequences of rising global rates of various non-communicable diseases (NCDs) [5].

Several chronic NCDs share inflammatory activities and metabolic impairment as common pathologies [6]. In the present study, we focused on two exemplary NCDs, (A) type 2 diabetes, as disease of low-grade chronic inflammation [7], and inflammatory bowel disease, as disease of high-grade chronic inflammation [8, 9]. For type 2 diabetes (T2D), it is known that a change of lifestyle and improvement of metabolic status can help to prevent disease onset [10]. However, between 1990 and 2021 the global age-standardized prevalence increased by 90.5% [11]. Similarly, such an increase in prevalence is found for inflammatory bowel disease (IBD), for which the global prevalence almost doubled between 1990 (3.7 million) and 2017 (6.8 million) [12]. IBD can be linked to metabolic inflammation [8] and dysbiosis of the gut microbiome [9], an alteration of the gut microbiome composition which can be influenced by diet, physical activity, and other lifestyle habits.

While the overarching link between changes in metabolic and lifestyle factors and NCDs is widespread, there are some critical aspects which, considering the continuously increasing disease prevalences, are not fully clear yet and need further investigation. Namely, the impact of biomedical and lifestyle factors on the timing of disease-onset as crucial determinant in prevention strategies, as well as the differentiated role of the gut microbiota when interacting with environmental factors and the host among diseased or healthy individuals.

For example, it is well established that a nutritious diet is one of the most important determinants of human health due to diverse protective properties [13, 14], yet its effective implementation for medical purposes remains challenging. One dietary pattern that has been in the focus of scientific attention is the Mediterranean Diet [15], which has formed the basis for modern dietary recommendations in many Western countries [16]. However, fundamental studies derive rather heterogeneous and even conflicting results [17], potentially caused by not considering the interactions between dietary compounds and the gut microbiota, even though it has been outlined that unfavorable dietary patterns reduce both the microbial diversity and variation [18]. Likewise, studies regarding the effects of physical activity on the onset of IBD yield inconsistent results. While some studies see an inverse relationship between physical activity and new-onset IBD in Crohn’s disease but not in ulcerative colitis [19], others recommend a dose–effect approach [20], and some find no effects at all [21]. Regarding physical activity and gut microbiome-related changes, results are just as non-uniform [22–24].

To shed light on these inconsistencies, our study aims to address the important aspect of disease timing while also considering lifestyle-gut microbiota-host interaction. Our research seeks to address the necessity for a multifaceted exploration. In more detail, we conduct an exploratory analysis to investigate the role of anthropometric, metabolic, and lifestyle factors in chronic NCDs, providing a comprehensive understanding of their impact. Our focus then extends to an in-depth examination of two specific NCDs (T2D and IBD) of the association between biomedical and lifestyle factors and age-at-disease-onset, describing the specific factors that influence disease initiation. Finally, we investigate how the identified important factors influence the abundance of gut microbiome genera and their differences between healthy individuals and those affected by either T2D or IBD.

To achieve these objectives, we employ data from three cohorts, comprising a collective sample size of over 1600 subjects from Northern Germany. The first cohort contains data including 11 NCDs, the other two cohorts are disease specific (diabetes and IBD). In total, our analysis includes 15 health-related factors for IBD and 18 for diabetes.

## Methods

### Study design and population

#### FoCus (cross-sectional exploration study)

We used comprehensive data from the German population-based Food Chain Plus (FoCus) cohort. A detailed description of the study cohort was published by Geisler et al. [25]. In brief, data collection was performed between 2011 and 2014 including a total of 1811 adults aged 18–83 years. Those were recruited through registration offices in the Kiel area in Northern Germany or in a case-specific manner through the obesity clinic of the University Medical Center Schleswig–Holstein (USKH) in Kiel where people with a BMI ≥ 30 kg/m^2^ and obesity-associated comorbidities are treated. Information about daily food intake, other lifestyle habits, and the presence and severity of chronic diseases were available from questionnaires. Furthermore, anthropometric and blood markers, bacterial abundances from 16S rRNA gut microbiota sequencing, and serum metabolite profiles were measured [25]. *N* = 1220 (female 716, male 504) cross-sectionally recruited participants (FoCus-CS) with complete anthropometric and lifestyle records were included in an exploratory analysis of the association between biomedical and lifestyle factors and the presence of 11 chronic NCDs: (1) obesity, (2) type 2 diabetes (T2D), (3) arterial hypertension, (4) hyperlipidemia, (5) chronic heart failure, (6) coronary artery disease, (7) inflammatory bowel disease (IBD), (8) rheumatoid arthritis, (9) psoriasis, (10) asthma, and (11) chronic bronchitis. Basic characteristics of the exploration cohort are provided in Table 1.

**Table 1:** Characterization of the FoCus-CS cohort

	**FoCus cross-sectional cohort**		
Subjects, *n*	1220		
Female sex, *n* (%)	716 (58.69)		
Age, years	54 [44-65]		
***Anthropometric measures***			
Height, cm	172 [167, 180]		
Weight, kg	76.3 [65.95, 88.3]		
BMI, kg/m^2^	25.4 [22.71, 28.72]		
***Non-communicable disease prevalence and age-at-onset***			
**Obesity**		**Inflammatory bowel disease** - Prevalence (95% CI) - Age at onset (among cases), years	0.06 (0.05, 0.08) 35 [23, 47]
- Prevalence (95% CI)	0.18 (0.16, 0.21)		
**Type 2 diabetes**			
- Prevalence (95% CI)	0.05 (0.03, 0.07)		
- Age-at-onset (among cases), years	57 [47.75, 63]		
**Arterial hypertension**		**Rheumatoid arthritis**	
- Prevalence (95% CI)	0.33 (0.30, 0.35)	- Prevalence (95% CI)	0.08 (0.06, 0.09)
- Age at onset (among cases), years	50 [42, 58]	- Age at onset (among cases), years	49 [35, 57]
**Hyperlipidemia**		**Psoriasis**	
- Prevalence (95% CI)	0.27 (0.20, 0.30)	- Prevalence (95% CI)	0.03 (0.02, 0.04)
- Age at onset (among cases), years	51 [42, 60]	- Age at onset (among cases), years	36 [22, 48.5]
**Chronic heart failure**		**Asthma bronchiale**	
- Prevalence, (95% CI)	0.02 (0.02,0.03)	- Prevalence (95% CI)	0.06 (0.05, 0.08)
- Age at onset (among cases), years	60 [46.75, 65.75]	- Age at onset (among cases), years	24 [12, 42.25]
**Coronary artery disease**		**Chronic bronchitis**	
- Prevalence, n (%)	0.04 (0.03, 0.06)	- Prevalence (95% CI)	0.04 (0.03, 0.05)
- Age at onset (among cases), years	58 [53, 64]	- Age at onset (among cases), years	40.5 [18.5, 48]

Data is presented as median [interquartile range], sex as number (total percentage), and categorical variables as prevalence (95% CI)

*Abbreviations*: *BMI* Body mass index

Based on the findings of this initial investigation, disease-specific in-depth analyses of (A) T2D and (B) IBD were conducted. We evaluated anthropometric, lifestyle-associated, metabolic, and disease-specific factors, summarized as biomedical and lifestyle factors, on their association with age-at-disease-onset and potential interaction with the gut microbiota in disease states. We employed two disease-specific study populations, which will be briefly described below:

#### FoCus-T2D (type 2 diabetes study)

Participants both from the cross-sectional and obesity-specific recruitment routes of the FoCus cohort described above have been stratified and selected based on their metabolic status: Participants who have stated a previous diagnosis of type 2 diabetes were defined as cases (*n* = 190). People, whose body mass index (BMI) was within normal range (18.5–25 kg/m^2^) [26], whose glucose, insulin, triglyceride, and C-reactive protein (CRP) levels were within normal range and who did not report a previously diagnosed cardio-metabolic disorder were defined as metabolically-healthy (*n* = 311) and served as a control group. Basic characteristics of this study population (FoCus-T2D, total: *n* = 514, female 334, male 167) are provided in Table 2.

**Table 2:** Characterization of the FoCus-T2D and IBD-KC cohorts

	**FoCus-T2D**			**IBD-KC**		
	**Cases**	**Controls**	***p***_***T2D***_^***a***^	**Cases**	**Controls**^**c**^	***p***_***IBD***_^***a***^
Subjects, *n*	197	317	-	623	487	-
Female sex, *n* (%)	112 (56.85)	234 (73.82)	8.17 × 10^−5^	418 (67.09)	284 (58.32)	3.2 × 10^−2^
Age, years	58 [48, 66]	51 [43, 64]	1.38 × 10^−5^	52 [39, 61]	49 [33, 64]	0.26
Age-at-disease-onset (average cases), years	50 [42, 58]	-	-	25 [19.5, 34]	-	-
*Anthropometry*						
Height, cm	172 [165, 180]	170 [166, 178]	< 10^−5^	170 [165, 176]	170 [164, 178]	0.62
Weight, kg	114.05 [90.85, 139.8]	64.5 [59.55, 71.4]	< 10^−5^	68 [59, 80]	70 [60.5, 80]	0.08
BMI, kg/m^2^	39.02 [31.11, 46.47]	22.32 [21.15, 23.51]	< 10^−5^	23.32 [20.96, 26.25]	23.95 [21.32, 26.82]	0.17
*Lifestyle*						
Diet						
- Total energy intake, kcal/day	2014 [1656, 2504]	2081 [1791, 2469]	0.1	2157 [1820, 2769]	2357 [1916, 2854]	2.61 × 10^−3^
- Scaled energy intake, kcal/kg BW/day	18.72 [13.96, 23.05]	32.1 [27.63, 38.31]	< 10^−5^	32.4 [26.8, 40.92]	33.47 [26.92, 43.65]	4.52 × 10^−2^
- HEI-EPIC, 0–100	52 [47, 56]	53 [48, 58]	0.06	48 [42, 54]	49 [43, 57]	2.69 × 10^−2^
- MDS, 0–9	4.5 [3, 5]	4 [3, 5]	0.96	4 [3, 5]	4 [3, 6]	0.16
Alcohol, g EtOH/day	0.92 [0.41, 2.57]	3.23 [1.35, 5.43]	< 10^−5^	3.4 [0.85, 9.94]	5.67 [1.42, 15.83]	2.96 × 10^−5^
Smoking						
- Smoker, prevalence (95% CI)	0.18 (0.13, 0.24)	0.15 (0.11, 0.19)	0.4	0.09 (0.07, 0.11)	0.12 (0.09, 0.15)	0.17
- Intensity (average smokers),*n* cigarettes/day	20 [6.5, 25]	10 [4.5, 15]	1.25 × 10^−3^	-	-	-
Activity						
- Everyday activity, h/week	14 [10, 21.5]	14.75 [9.62, 22]	0.62	13 [8, 20.88]	11.5 [6.5, 21]	5.14 × 10^−3^
- Sports, h/week^b^	1.5 [0, 4]	4.5 [2.62, 7.5]	< 10^−5^	3.25 [1.27, 6]	4 [1.5, 6.5]	0.07
- TV watching, h/week^b^	21 [14, 35]	14 [10.5, 21]	< 10^−5^	8.5 [4, 14]	9 [4, 14]	0.31
Sleep, h/24 h	8 [7, 9]	7 [7, 8]	3.37 × 10^−3^	7.63 [7, 8.5]	7.75 [7, 8]	0.75
Sleep, h/daytime	0.5 [0, 1]	0 [0, 0.25]	< 10^−5^	0 [0, 0.5]	0 [0, 0.33]	4.99 × 10^−3^
*Gut microbiota*						
Shannon index	2.48 [2.25, 2.69]	2.62 [2.41, 2.79]	2.49 × 10^−4^	2.16 [1.89, 2.39]	2.27 [2.07, 2.43]	< 10^−5^
Chao1 index	56 [49, 66]	58 [53, 63]	0.71	42 [35, 49]	46 [41, 52]	< 10^−5^

Data is displayed as median [Interquartile Range]

*Abbreviations:* *IBD* Inflammatory bowel disease, *BMI* Body mass index, *BW* Body weight, *HEI-EPIC* Healthy Eating Index determined from EPIC-FFQ, *MDS* Mediterranean Diet Score, *EtOH* Ethanol

^a^Chi-square test for categorical variables, Wilcoxon-test for continuous variables

^b^These variables were differently documented between cohorts

^c^Controls were recruited as first degree relatives from cases without IBD

#### IBD-KC (Inflammatory bowel disease study)

The Inflammatory Bowel Disease Kindred cohort (IBD-KC) is a family-based cohort specifically focused on inflammatory bowel disease. Briefly, the IBD-KC is a nationwide prospective study initiated in 2013 in Kiel, Germany, and ongoing since then, with a current involvement of 1715 study participants. We used the cohort as a case–control study design, index patients as cases and relatives as healthy controls. At baseline, a total of 1110 participants from 503 families with complete dietary records and clinical variables (see Table 2) were used for the current analyses.

Recruitment of IBD patients was carried out through treating physicians, clinics, study flyers, letters, and information disseminated by patient organizations like the German Crohn’s Disease/Ulcerative Colitis Association. Questionnaires were filled out in a self-reported manner and in addition by their treating physician.

**Fig. 1:**
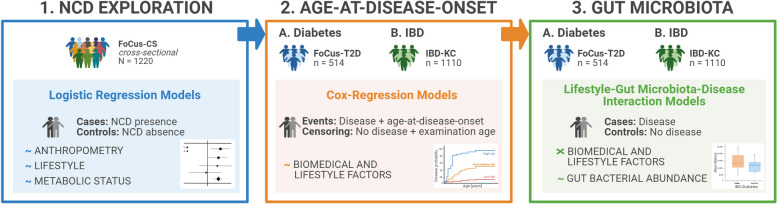
Study design and populations. Displayed is the study design divided into three sections: Initially, an exploratory analysis regarding the association of biomedical and lifestyle factors with the presence of 11 common non-communicable diseases has been performed in the cross-sectional Kiel FoCus cohort (FoCus-CS) using logistic regression models. Afterwards, two disease-specific cohorts, FoCus-T2D and IBD-KC, were utilized for an in-depth analysis of disease probability of type 2 diabetes (T2D) and inflammatory bowel disease (IBD), respectively. Here, the association between biomedical and lifestyle factors and age-at-disease-onset has been evaluated using Cox-PH-regression models and time-to-event analysis. Finally, we analyzed the effects of biomedical and lifestyle factors on the abundance of genera in the gut microbiota, regarding differences between cases and controls, applying MaAsLin2 (Microbiome Multivariable Associations with Linear Models) [27]. Abbreviations: NCD, non-communicable disease; T2D, Type 2 diabetes; IBD, inflammatory bowel disease

Figure 1 provides an overview of the study design and respectively used study populations.

### Disease-specific markers

#### FoCus

Blood samples from each participant were collected at the study site by trained medical staff. Clinical metabolic markers were quantified at the central laboratory of the UKSH, Kiel; GLP-1 was measured at the Institute of Diabetes and Clinical Metabolic Research using a commercial ELISA kit (Mercodia—No. 10–1278-01). Homeostatic Model Assessment for insulin resistance was calculated from glucose and insulin values (HOMA-IR = (fasting glucose × fasting insulin/ 405)).

#### IBD-KC

The treating physicians were responsible for conducting blood sampling. The collected tubes were dispatched to the study laboratory located at the Institute of Clinical Molecular Biology in Kiel. The measurement of calprotectin was performed utilizing Bühlmann fCAL, with reference to external laboratory findings.

### Diet

#### Data collection and accuracy validation

The habitual dietary intake was captured using the validated semi-quantitative 12-month retrospective food frequency questionnaire by the European Investigation into Cancer and Nutrition protocol from the German Institute of Human Nutrition (EPIC-FFQ, version 1) [28]. The EPIC-FFQ consists of questions about the consumption frequency and average portion size of 143 food items. Using the questionnaire data, subsequent calculations with the “EPICsoft” software result in information on the total caloric, food and water intake, as well as data of the micro- and macronutrient, single food item, or food group intake [29]. Data accuracy is ensured by two-step validation: An initial validation, which has previously been described by Nöthlings et al. [30] and an additional internal data check via unannounced 24-h-dietary recall in 10% of subjects by a trained dietician.

#### Diet quantity

Two measures were used for the evaluation of diet quantity: (1) the total caloric intake taken from the EPIC-FFQ results [kilocalories (kcal)/day] and (2) a scaled caloric intake as a measure of consumed calories in relation to body weight [kcal/kg body weight/day].

#### Diet quality

For a precise assessment of the diet quality, an adapted form of the Healthy Eating Index (HEI) [31] and Mediterranean Diet Score (MDS) [32] were calculated from the above described EPIC-FFQ. Detailed descriptions of score constitutions along with calculation protocols are provided in the Additional file 1.

### Statistical analysis

Statistical analysis and data visualization was performed with R (Windows: R Core Team, 2022 version 4.1.2, Ubuntu Jammy: R version 4.3.0), R Studio (Windows: Version 1.4.1717, Ubuntu Jammy: “Desert Sunflower” 2023.09.1), as well as using Jupyter notebook (version 6.4.8 of notebook server and Python (version 3.10.12)). Univariate comparison of continuous variables was performed using Mann–Whitney *U*-test (two independent groups) or Kruskal–Wallis test (> two independent groups). Correlation of two continuous variables was performed using Spearman’s rank correlation tests. When using the IBD-KC family-based cohort, it is always adjusted for the family effect by considering the family ID as a random effect.

#### Exploratory analysis of phenotype and biomedical and lifestyle factors with NCDs

For each NCD (considered as independent variable: absence/presence), unadjusted (univariable) and adjusted (multivariable) logistic regression models were formed and evaluated. Biomedical and lifestyle factors served as dependent variables (26 variables in total). Adjustments were made for sex and BMI class (for details see the Additional file 1). Forest plots of odds ratios (95% confidence intervals) were generated for every NCD with a *p*-value < 0.1. Furthermore, Spearman’s correlation analysis within cases was used to evaluate the association between age-at-disease onset and the assessed factors. Findings are displayed as correlation heatmap. Plots were generated using the R ggplot2 package (version 3.4.3).

#### Association between biomedical and lifestyle factors and age-at-disease-onset

For each biomedical and lifestyle factor (considered as an independent variable), we conducted a Cox proportional hazards regression analysis (Cox-PH-regression). Age-at-disease-onset (for cases) or current age at baseline (age at examination for healthy controls) served as dependent variable, disease status was considered as censoring indicator, and the model was adjusted for sex and BMI class. Using a likelihood ratio test, biomedical and lifestyle factors which significantly improve the model were determined by comparing the full model, including the biomedical or lifestyle factor, with a reduced model, excluding the biomedical or lifestyle factor. Kaplan–Meier curves were constructed to estimate survival times for biomedical and lifestyle factors that showed *p*-values < 0.1. For graphical visualization and hazard ratio calculation, all continuous variables are categorized in terciles, and the reference category is chosen as “low”. For more details, see the Additional file 1. The R survival package (version 3.5–7) and the coxph function were utilized for this analysis.

#### Role of biomedical and lifestyle-gut microbiota interactions in diabetes and IBD

To investigate interactions between biomedical and lifestyle factors and the gut microbiome composition, we used the R package Maaslin2 (Microbiome Multivariable Association with Linear Models) [27], for details see the Additional file 1. Here, the disease state (binary) served as an independent variable contributing to the interaction term, which also incorporated each previously identified significant biomedical or lifestyle factor and variables we adjusted for. The dependent variable was a genus within the microbiome. For the IBD-KC family cohort, the family structure is considered as a random effect.

Initially, the model was fitted for each entire dataset (IBD-KC and FoCus-T2D). To evaluate if there are differences between cases and controls in the effect of biomedical and lifestyle factors on the abundance of one genus, Maaslin2 was separately run for the data for cases and controls within each cohort. In these separate runs, only the respective biomedical or lifestyle factor was included. Whereas IBD is widely recognized for its close association with inflammation in the microbiome, diabetes is primarily linked to metabolic dysfunction. Consequently, we anticipated discovering more significant associations in the context of IBD. Hence, we explored three associations per factor for IBD and up to five associations per factor for diabetes. The selection of associations is based on the smallest false discovery rate (FDR)-corrected *p*-values, along with additional significant associations within diabetes cases. Model coefficient values (effects size) and corrected *p*-values were compared between all, only cases, and only controls, focusing on associations that were significant for cases but not controls and vice versa.

## Results

### Characterization of the exploratory study population (FoCus-CS)

Differences in anthropometry, lifestyle, microbial diversity, and metabolic blood markers between 11 common NCDs and non-affected subjects were assessed using the German population-based FoCus cohort (FoCus-CS, *N* = 1220). Characterization of this study population, including disease prevalence of the assessed NCDs, is displayed in Table 1.

### Exploratory analysis of metabolic phenotype and biomedical and lifestyle factors in association with common chronic non-communicable diseases

**Fig. 2:**
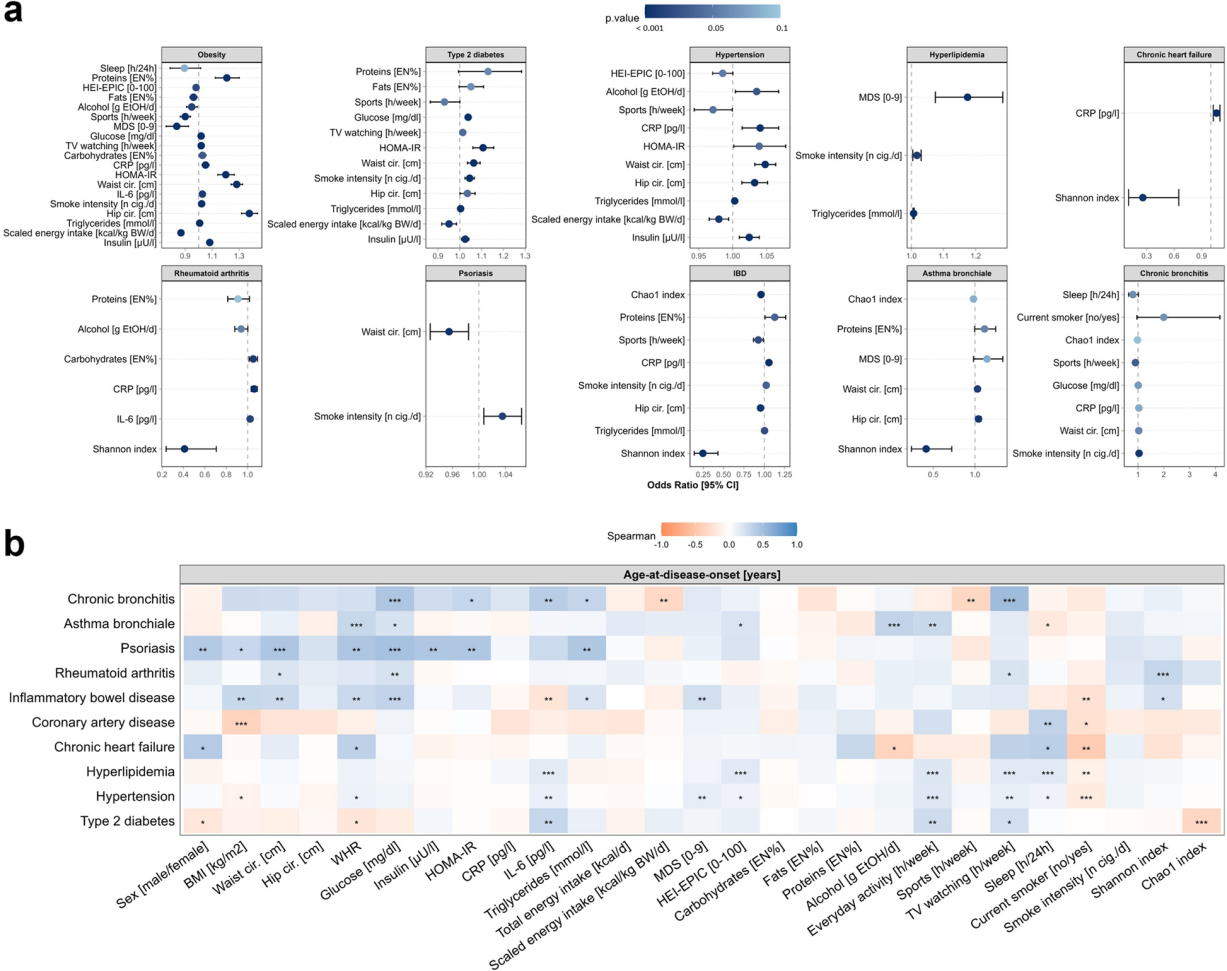
Exploratory analysis of phenotype and biomedical and lifestyle factors in association with common NCD. Presented are results of the exploratory analysis of 10 NCDs and respective age-at-disease-onset in relation to selected biomedical and lifestyle factors. **a** Multiple logistic regression models (disease presence/absence) adjusted for age, sex (obesity), and additionally obesity status (all other NCDs). Odds ratios (OR) estimated from factors with *p* < 0.1 (dot color indicating *p*-value). Due to very high ORs, associations with waist-to-hip ratio are not displayed. **b** Age-at-disease-onset for cases of the assessed NCDs was analyzed in association with the introduced factors using Spearman’s rank correlation tests. Presented is a heatmap of Spearman’s rank correlation coefficients with asterisks denoting significance levels (*p* < 0.01 = ***, *p* < 0.05 = **, *p* < 0.1 = *). Abbreviations : NCD, non-communicable disease; MDS, Mediterranean Diet Score; HEI-EPIC, Healthy Eating Index determined from EPIC-FFQ; HOMA-IR, Homeostatic Model Assessment of Insulin Resistance; CRP, C-reactive protein; IL, interleukin; IBD, inflammatory bowel disease; BMI, Body Mass Index; WHR, waist-to-hip ratio

In an exploratory analysis, we assessed the association of biomedical and lifestyle factors (26 factors/markers in total) with the prevalence of 11 common NCDs using logistic regression models, utilizing data from the cross-sectional FoCus cohort (see Table 1). Unadjusted logistic regression analysis of each NCD is provided in the Additional file 1: Table S3 and Fig. S1. Due to initial differences in NCD prevalence with age, sex, and obesity, multiple logistic regression models shown here were adjusted for these three factors. Figure 2a displays relevant factors (*p* < 0.1) for each NCD. Displayed are forest plots of odds ratios (OR), 95% confidence intervals (CI), and *p*-values given by dot color, while exact ORs, CIs, and *p*-values are provided in the Additional file 1: Table S4. Since here was only a trend in triglyceride alteration (OR = 1.003 [0.999–1.006], *p* = 0.09) detectable with coronary artery disease but no other relevant association, this disease is not displayed in the figure.

#### Metabolic phenotype

As expected, this analysis shows that an altered metabolic phenotype and blood profile are linked to obesity, T2D and arterial hypertension. Increased waist circumferences (obesity: OR = 1.28 [1.24–1.32]; T2D: OR = 1.06 [1.03–1.09]; hypertension: OR = 1.05 [1.03–1.07]) and waist-to-hip-ratio (WHR, obesity: OR = 4.22 × 10^7^ [2.67 × 10^7^–6.69 × 10^8^]; T2D: OR = 594 [21.74–16,265]; hypertension: OR = 230 [28.49–1864]) are found in people affected by at least one of these diseases. ORs associated with a one-unit-increase in WHR are very high, due to the small range of observed WHRs in the sample (Median = 0.87 [0.79–0.94]). In accordance, metabolic blood markers glucose, insulin, and HOMA-IR also exhibit increased values at metabolic disease presence (see Fig. 2a). While alterations in glucose markers are limited to metabolic diseases, alterations in anthropometry are present in IBD (WHR: OR = 18.18 [0.75–438]) and asthma (waist measure: OR = 1.03 [1.01–1.05]). Of note, psoriasis is the only disease displaying an inverse association with waist circumference (OR = 0.95 [0.92–0.98]).

#### Biomarkers of metabolic inflammation

To evaluate the level of metabolic inflammation, we determined triglyceride serum concentrations as a marker for metabolic disturbances and CRP levels as an inflammatory marker. Triglyceride levels are altered in some of the assessed NCDs displaying mildly increased odds ratios: obesity (OR = 1.01 [1.01–1.01]) and hyperlipidemia (OR = 1.01 [1.01–1.01]). Similarly, chronic inflammatory activity is present in obesity (OR = 1.05 [1.02–1.08]), hypertension (OR = 1.04 [1.01–1.07]), chronic heart failure (OR = 1.06 [1.02–1.1]), rheumatoid arthritis (OR = 1.06 [1.03–1.09]), and IBD (OR = 1.06 [1.03–1.09]).

#### Diet

Dietary differences towards reduced dietary quality, increased caloric quantity, and shift in macronutrient composition were detectable in metabolic diseases obesity (MDS: OR = 0.84 [0.76–0.93]; HEI-EPIC: OR = 0.98 [0.96–1], scaled energy intake: OR = 0.86 [0.85–0.89]; carbohydrate intake: OR = 1.03 [1,1.06]; protein intake: OR = 1.21 [1.12–1.3]; fat intake: OR = 0.96 [0.93–0.99]) and T2D (scaled energy intake: OR = 0.95 [0.92–0.98). A similar observation regarding caloric quantity can be seen in arterial hypertension (scaled energy intake: OR = 0.98 [0.97–0.99]). A uniform trend of increased dietary protein intake can be seen in metabolic diseases, IBD (OR = 1.13 [1.01–1.26]), and asthma (OR = 1.12 [1–1.25]). In return, a trend of lower protein intake (OR = 0.91 [0.81–1.02]), but higher carbohydrate intake (OR = 1.05 [1.01–1.09]) is present in rheumatoid arthritis. Evaluation of the MDS indicates higher adherence to a Mediterranean-style diet in people with hyperlipidemia (OR = 1.17 [1.07–1.28]). Dietary intake does not display potent associations of disease state with heart failure, coronary artery disease, psoriasis, asthma, and bronchitis.

#### Physical activity and TV consumption

For the evaluation of physical activity, everyday tasks representative for moderate and sports representative for vigorous activities were available. This analysis revealed no associations with everyday activities but decreased rates of vigorous sports activity in obesity (OR = 0.9 [0.86–0.94], hypertension (OR = 0.97 [0.94–1]), IBD (OR = 0.93 [0.87–0.99]), and chronic bronchitis (OR = 0.9 [0.81–0.99]). In addition to decreased sports activity, TV consumption was increased in obesity (OR = 1.02 [1.01–1.03]) but not hypertension, IBD, or bronchitis.

#### Sleep duration

Aside from diet and physical activity, it is now well known that the duration and quality of sleep is another important determinant for the preservation of health. Accordingly, altered sleep duration, which was available for our analysis, was present in obesity (OR = 0.9 [0.79–1.02]) but no other disease.

#### Smoking

Cigarette smoking is a risk for numerous of the observed NCDs, and while rates of smokers do not display significant differences among disease presence or absence, diseased subjects present higher cigarette consumption per day, e.g., in T2D (OR = 1.05 [1.03–1.07], IBD (OR = 1.02 [1–1.04]), and chronic bronchitis (OR = 1.04 [1.01–1.07]).

#### Alcohol

A higher alcohol consumption is displayed in people affected by arterial hypertension (OR = 1.04 [1.01–1.07]). Yet, most of the evaluated diseases display either no or even an opposite association with alcohol intake.

#### Microbial diversity

A decreased gut microbial diversity has been linked to disease etiology of various diseases and accordingly can be observed in several of the evaluated NCDs, like IBD (Shannon: OR = 0.25 [0.14–0.44]; Chao1: OR = 0.96 [0.94–0.98]), rheumatoid arthritis (Shannon: OR = 0.41 [0.24–0.71]), chronic heart failure (Shannon: 0.26 [0.1–0.66]), and asthma (Shannon: OR = 0.41 [0.24–0.71]) within our study. However, neither Shannon nor Chao1 indices display significant alternations in comparison to the respective control group for obesity, T2D, hypertension, hyperlipidemia, coronary artery disease, psoriasis, and chronic bronchitis.

The second part of this investigation involves a Spearman correlation analysis, examining the introduced biomedical and lifestyle factors in connection to age-at-disease-onset within disease cases. As presented in Fig. 2b, biomedical and lifestyle factors differently associate with the disease-onset-age for each NCD. Overall, this analysis reveals that while some of the assessed factors initially have not shown differences between disease presence or absence, they still demonstrate correlations with the age-at-disease onset, emphasizing their relevance in the disease course, onset, and prevention. For instance, this can be seen for type 2 diabetes, where the gut microbial diversity (represented through the Chao1 index) was no risk factor for the disease, but a significant association with age-at-disease-onset was found (*r*_S_ =  − 0.355, *p* = 5.86 × 10^−3^). Our analysis further displays that in IBD, there is an association between reduced BMI as well as WHR and earlier age-at-disease-onset (BMI: *r*_S_ = 0.292, *p* = 1.2 × 10^−2^; WHR: *r*_S_ = 0.305, *p* = 8.67 × 10^−3^), while no differences in these anthropometric factors can be seen with disease presence/absence.

### Characterization of disease-specific cohorts

Additionally, we performed an in-depth analysis of the age-at-disease-onset for two highly prevalent NCDs related to metabolic inflammation and alterations of the gut microbiota—type 2 diabetes and inflammatory bowel disease. T2D primarily involves metabolic dysfunction, while IBD pertains more to chronic inflammation. For both diseases, alterations of the gut microbiota are well established, in contrast to the multitude of other assessed NCDs, where such alterations could not be clearly outlined. In-depth analysis was performed using two disease-specific cohorts for (A) type 2 diabetes (FoCus-T2D, *N* = 514) and (B) inflammatory bowel disease (IBD-KC, *N* = 1110). These cohorts consist of disease cases and free-of-target-disease control subjects. The IBD-KC cohort is a family-based dataset, here used as a case–control study design, where controls were recruited as relatives from index IBD cases. A comprehensive characterization of study populations, including comparison between cases and control subjects is given in Table 2; information on disease-specific factors and microbial beta diversity can be found in the Additional file 1: Tables S4A + B and Fig. S2, respectively.

### Association between biomedical and lifestyle factors and age-at-disease-onset in T2D and IBD

**Fig. 3:**
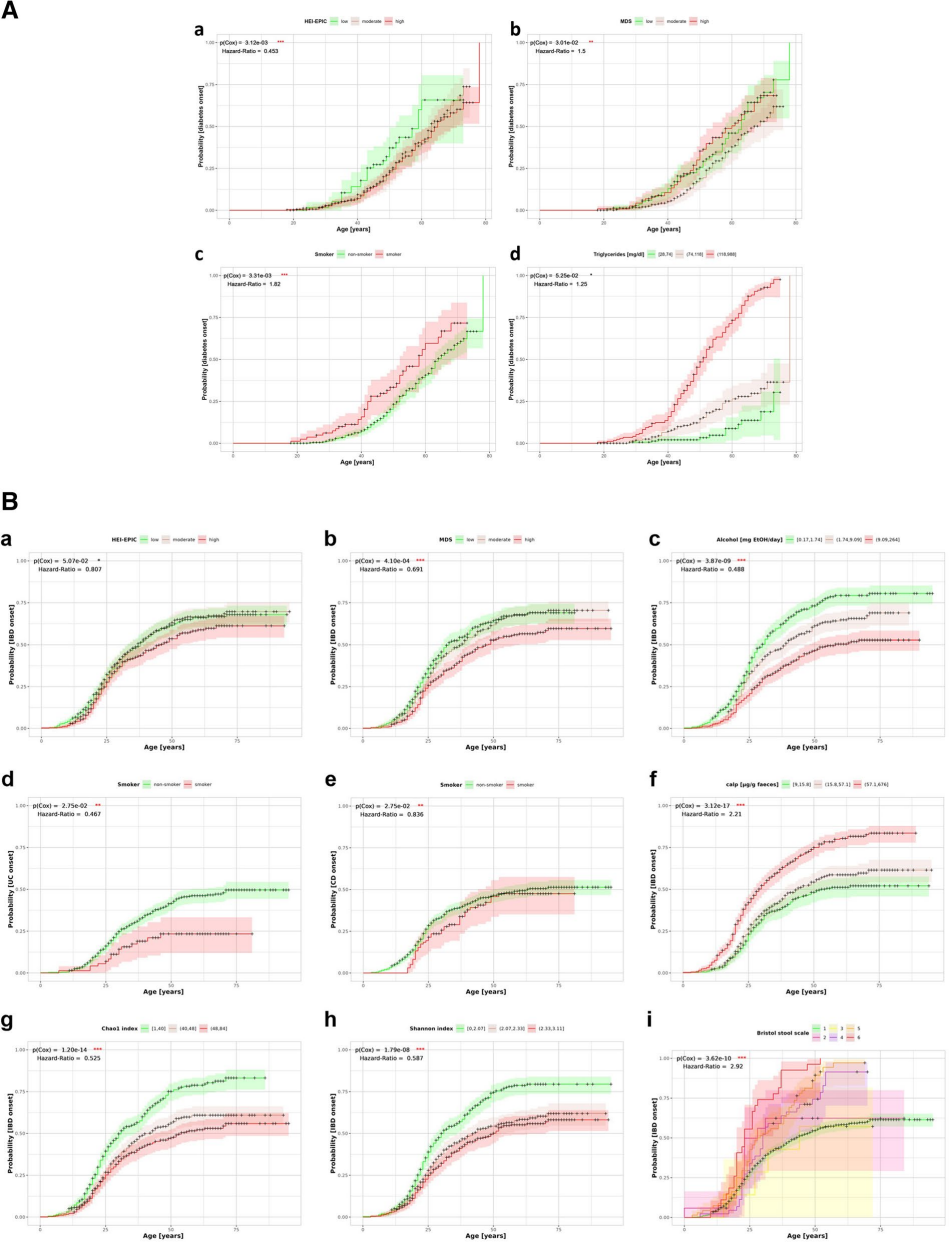
**a** Kaplan-Meier survival plots for biomedical and lifestyle factors and type 2 diabetes risk. This figure displays Kaplan-Meier survival plots for biomedical and lifestyle factors (*a*) HEI-EPIC, (*b*) MDS, (*c*) Smoker, and (*d*) Triglycerides in relation to the risk of T2D, assessed based on age-at-disease-onset (diabetes cases) and censored age-at-examination (control subjects). Color-coded levels within each parameter signify various groups or levels. Asterisks and color denote significance levels (*p*_*Cox*_< 0.01 = ***, *p*_*Cox*_< 0.05 = **, *p*_*Cox*_ < 0.1 = *), additionally the total hazard ratio (HR) between the lowest and the highest level for each biomedical or lifestyle factor is given from the Cox regression models. Abbreviations: HEI-EPIC, Healthy Eating Index adapted to EPIC Food Frequency Questionnaire data; MDS, Mediterranean Diet Score. **b** Kaplan-Meier survival plots for biomedical and lifestyle factors and IBD risk. This figure displays Kaplan-Meier survival plots for biomedical and lifestyle factors (*a*) HEI-EPIC, (*b*) MDS, (*c*) Alcohol, (*d*) Smoker UC, (*e*) Smoker CD, (*f*) Calprotectin, (*g*) Chao1 index, (*h*) Shannon index, and (*i*) Bristol stool scale in relation to the risk of IBD, assessed based on age-at-disease-onset (IBD cases) and censored age-at-examination (control subjects). Color-coded levels within each parameter signify various groups or levels. Asterisks and color denote significance levels (*p*_*Cox*_< 0.01 = ***, *p*_*Cox*_< 0.05 = **, *p*_*Cox*_ < 0.1 = *), additionally the total hazard ratio (HR) between the lowest and the highest level for each biomedical or lifestyle factor is given from the Cox regression models. Abbreviations: MDS, modified Mediterranean Diet Score; UC, ulcerative Colitis; CD, Crohn’s disease; calp: calprotectin

Each biomedical lifestyle factor (independent variable) was investigated using individual Cox proportional hazards regression, adjusting for sex and BMI class (for details see “Statistical analysis”), with age-at-disease-onset (for cases) or censored age-at-examination (for controls) as dependent variables and disease status as censoring indicator. Continuous biomedical and lifestyle factors were categorized into either terciles or deciles. Presented are biomedical and lifestyle factors and disease specific markers, relevant for T2D (Fig. 3a) and IBD (Fig. 3b). The Kaplan Meier survival plots depict the probability of disease occurrence in relation to age, with each evaluated factor considered individually. In both conditions, interesting associations (*p*-value < 0.1 when comparing the full model with the reduced model) emerged for various lifestyle factors related to nutrition, gut microbiome, and anthropometrics. Hazard ratios and confidence intervals for those lifestyle factors can be found in the Additional file 1: Tables S6A + B. It should be noted that the data used are cross-sectional, with both disease status and biomedical and lifestyle factors collected at baseline. That is why only hazard ratios (reference group is “low” for all factors), but no absolute age-dependent prevalences can be estimated and no statements about causality can be made, interpretation will focus on correlation and association.

For T2D, the association between age-at-disease-onset and BMI has been evaluated in a simple model only adjusted for the participants’ sex. This displays very low risk among under- and normal weight subjects and monotonously earlier onset from overweight to obesity III. Due to that association, further evaluation of biomedical and lifestyle factors was performed not only adjusting for sex but also for BMI class. Associations with *p*-values < 0.1 can be found for the diet quality assessed by the HEI-EPIC and MDS, current smoking status and triglyceride serum levels, while physical activity, sleep duration, microbial diversity, inflammatory, and glucose metabolism markers could not directly be linked to age-at-diabetes-onset. In more detail, the HEI-EPIC is a measure for an overall quality diet. People with high diet quality, indicated by the HEI-EPIC level, show a delay in T2D onset indicated by a hazard ratio of 0.64 [0.396–1.034] (low-moderate) and 0.453 [0.284–0.722] (low–high) (see Fig. 3a (a)). The MDS, however, does not show such clear association with only slightly later T2D onset in people with moderate (HR = 0.947 [0.669–1.340]), but not high MDS values (HR = 1.495 [1.025–2.180]) within our study population (see Fig. 3a (b)). In this context, it should be considered that dietary recommendations for individuals with diabetes closely resemble a Mediterranean diet [31]. Consequently, the assessment of the Mediterranean Diet Score (MDS) may be susceptible to bias, a problem of the case–control study design, where no information on dietary habits before disease onset is available. Consistently, within our study population, HEI-EPIC levels have a similar distribution in diabetes cases and controls, while a larger proportion of cases (28.93%) were assigned to the high level of MDS compared to controls (18.93%). Cigarette smoking is also associated with the probability of developing diabetes in this population. Smokers experience an approximately 4 years earlier median age-at-disease-onset (see Fig. 3a (c)). Finally, as seen in Fig. 3a (d), elevated blood triglyceride levels may also be of interest when considering earlier diabetes onset (low-moderate HR = 1.166 [0.579–2.349], low–high HR = 1.249 [0.647–2.412], *p* = 0.05).

For IBD, factors associated with age-at-disease-onset with *p*-values < 0.1 include the diet quality (HEI-EPIC and MDS), alcohol consumption, current smoking status, microbial diversity markers (Shannon and Chao1 index), as well as disease-specific markers like calprotectin and the Bristol stool scale. Again, the case–control study design does not allow estimation of age-specific prevalences, and interpretation is done with a focus on relative disease risk. Concerning diet, individuals with a high HEI-EPIC score experience a median age-at-disease-onset 8 years later than those with a low diet score (see Fig. 3b (a)). Additionally, the MDS score demonstrates an even stronger delay in disease onset by 16 years (low–high HR = 0.691 [0.567–0.841]) as illustrated in Fig. 3b (b). This positive correlation between a healthy diet and a lower likelihood of developing IBD at any age is supported by a consistent decrease in the hazard ratio with increasing diet scores. A less intuitive result pertains to the association between age-at-disease-onset and alcohol intake (Fig. 3b (c)). Individuals in this study with a higher alcohol intake show a decreased risk of developing IBD with an HR of 0.488 [0.397–0.599]. No significant associations were found for the impact of physical activity (sports, watching TV, and everyday activity), sleep, and total or scaled energy intake and age-at-disease-onset. Another interesting finding (Fig. 3b (d + e)) is that for UC, smoking correlates with a delayed age-at-disease-onset (HR = 0.467 [0.277–0.785]). However, for CD, there is no significant association with current smoking habits (HR = 0.836 [0.604–1.158]). As anticipated, participants with greater gut microbiota diversity experience a delayed age-at-disease-onset compared to those with lower diversity (Fig. 3b (g + h)). The hazard ratio for both the Chao1 and the Shannon index is consistently below one, indicating a significantly decreased risk of developing IBD associated with a higher gut diversity in this study population. As depicted in Fig. 3b (i), individuals from the analyzed families with diarrhea-like stool types have a higher IBD hazard (1 < HR < 2.9) compared to those with normal stool types. Another disease-specific marker, calprotectin (Fig. 3b (f)), indicates that individuals from this cohort with high calprotectin values are more likely to develop IBD (low–high HR = 2.21 [1.819–2.691]).

When examining the Kaplan–Meier plots for T2D (Fig. 3a) and IBD (Fig. 3b), it is noteworthy that the curves for diabetes exhibit a linear increase in disease prevalence with age. In contrast, the relationship is more accurately characterized by an exponential function for IBD. This signifies that hardly any new-onset IBD cases were observed in the older age groups in this study, a pattern not observed in diabetes.

### Role of biomedical and lifestyle factors-gut microbiota interactions in diabetes and IBD

Recognizing inconsistencies in previously described connections between certain biomedical and lifestyle factors and the composition of the human gut microbiota, we aim to delve deeper into understanding potential differences between diseased (T2D and IBD) and healthy individuals regarding the impact of biomedical and lifestyle factors on the abundance of genera in the gut microbiome. We use 16S abundance data at genus level and the previously described pre-processing steps (see “Statistical analysis”) for the IBD-KC and FoCus-T2D cohorts.

Our results, presented in Tables 3 and 4, highlight significant associations (FDR-corrected *p*-value < 0.05) between each biomedical or lifestyle factor and genus. To gain a comprehensive understanding, we initially examined cases and controls together to identify interacting factors. Subsequently, based on the differences found in microbial diversity between T2D and IBD patients and their respective control groups (see the Additional file 1:Fig. S2), we conducted separate analyses for cases and controls, replacing the interaction term with the biomedical or lifestyle factor alone. Distinct patterns emerged in both cohorts; specific genera exhibited significant associations with biomedical and lifestyle factors for cases but not controls.

**Table 3:** Interaction analysis of selected biomedical and lifestyle factors with T2D on the abundance of gut bacterial genera

Biomedical and lifestyle factors	Genus	Coef_interaction_	*p*_interaction_	Coef_cases_	*p*_cases_	Coef_controls_	*p*_controls_
**Sex**	*Anaerovorax*	− 0.43	2.63 × 10^−5^	0.4	0.72	− 0.39	0.3
	*Barnesiella*	− 0.4	1.15 × 10^−4^	− 0.86	0.09	− 0.24	0.68
	*Faecalibacterium*	− 0.25	1.89 × 10^−4^	0.33	0.72	0.2	0.59
**BMI**	*Alistipes*	− 0.31	< 10^−5^	− 0.22	0.25	− 0.004	n.s
	*Barnesiella*	− 0.45	< 10^−5^	0.16	0.72	− 0.05	n.s
	*Acidaminococcus*	0.82	1.09 × 10^−5^	0.25	0.79	0.06	n.s
	*Catabacter*	− 0.58	4.18 × 10^−5^	− 0.65	1.39 × 10^−2^	0.14	n.s
	*Halomonas*^*a*^	0.84	1.16 × 10^−2^	1.07	3.44 × 10^−3^	-	-
**HEI-EPIC**	*Barnesiella*	− 0.49	< 10^−5^	0.12	0.89	− 0.003	0.99
	*Alistipes*	− 0.26	1.16 × 10^−5^	0.03	0.89	− 0.05	0.86
	*Anaerovorax*	− 0.41	1.16 × 10^−5^	− 0.17	0.89	− 0.09	0.86
	*Mitsuokella*^*a*^	0.84	2.85 × 10^−3^	1.15	7.57 × 10^−3^	-	-
**MDS**	*Barnesiella*	− 0.49	< 10^−5^	− 0.02	0.96	− 0.05	0.94
	*Anaerovorax*	− 0.43	1.26 × 10^−5^	− 0.22	0.46	− 0.03	0.94
	*Acidaminococcus*	0.75	2.58 × 10^−5^	0.04	0.96	0.03	0.94
	*Mitsuokella*^*a*^	0.86	7.5 × 10^−5^	1.21	2.15 × 10^−2^	-	-
**Triglycerides**	*Oscillibacter*	− 0.36	< 10^−5^	− 0.36	0.07	0.05	0.71
	*Acidaminococcus*	0.66	< 10^−5^	0.17	0.72	0.03	0.55
	*Alistipes*	− 0.28	1.66 × 10^−5^	− 0.17	0.31	− 0.07	0.67
**Smoker**	*Bilophila*	0.35	3.63 × 10^−5^	1.31	6.6 × 10^−3^	− 0.23	0.92
	*Alistipes*	− 0.25	7.31 × 10^−4^	− 0.65	0.14	− 0.01	0.97
	*Barnesiella*	− 0.29	4.83 × 10^−2^	− 0.38	0.73	0.1	0.93

*Abbreviations*: *BMI* Body mass index, *HEI-EPIC* Healthy Eating Index determined from EPIC-FFQ, *MDS* Mediterranean Diet Score

^a^Genus abundance did not reach initial thresholds in the control group and therefore was not analyzed

**Table 4:** Interaction analysis of selected biomedical and lifestyle factors with IBD on the abundance of gut bacterial genera

Biomedical and lifestyle factors	Genus	Coef_interaction_	*p*_interaction_	Coef_cases_	*p*_cases_	Coef_controls_	*p*_controls_
**Sex**	*Veillonella*	0.75	< 10^−5^	0.13	0.48	0.007	0.98
	*Oscillibacter*	− 0.27	< 10^−5^	0.17	0.02	0.06	0.27
	*Clostridium_XlVa*	0.32	< 10^−5^	0.09	0.25	0.02	0.85
**BMI**	*Oscillibacter*	− 0.34	< 10^−5^	− 0.05	0.72	− 0.01	0.87
	*Veillonella*	0.72	< 10^−5^	− 0.2	0.31	− 0.23	0.23
	*Clostridium_IV*	− 0.35	< 10^−5^	− 0.09	0.58	− 0.01	0.91
**HEI-EPIC**	*Oscillibacter*	− 0.3	< 10^−5^	0.11	0.29	0.02	0.86
	*Veillonella*	0.72	< 10^−5^	− 0.08	0.74	0.15	0.45
	*Clostridium_IV*	− 0.33	< 10^−5^	− 0.04	0.76	− 0.05	0.83
**MDS**	*Oscillibacter*	− 0.27	< 10^−5^	0.082	0.29	0.076	0.25
	*Subdoligranulum*	− 0.3	< 10^−5^	− 0.08	0.46	− 0.04	0.71
	*Clostridium_IV*	− 0.32	< 10^−5^	− 0.08	0.46	− 0.12	0.24
**Alcohol**	*Oscillibacter*	− 0.21	< 10^−5^	− 0.09	0.47	− 0.08	0.37
	*Odoribacter*	− 0.18	0.019	− 0.11	0.51	− 0.07	0.61
	*Lactobacillus*	0.21	0.019	0.2	0.09	0.09	0.82
**Smoker**	*Oscillibacter*	− 0.22	< 10^−5^	− 0.21	0.01	− 0.13	0.12
	*Intestinimonas*	− 0.31	< 10^−5^	− 0.37	0.004	0.007	0.93
	*Catabacter*	− 0.39	< 10^−5^	− 0.44	0.01	− 0.06	0.70
**Bristol Stool Scale**	*Oscillibacter*	− 0.38	< 10^−5^	− 0.25	< 10^−5^	− 0.1	0.47
	*Clostridium_IV*	− 0.42	< 10^−5^	− 0.35	< 10^−5^	0.01	0.95
	*Subdoligranulum*	− 0.35	< 10^−5^	− 0.21	0.02	− 0.07	0.67
**Calprotectin**	*Flavonifractor*	0.2	< 10^−5^	0.18	0.01	0.02	0.92
	*Clostridium_XlVa*	0.21	< 10^−5^	0.13	0.10	0.008	0.92
	*Lactobacillus*	0.48	< 10^−5^	0.44	0.004	− 0.21	0.77

*Abbreviations*: *BMI* Body mass index, *HEI-EPIC* Healthy Eating Index determined from EPIC-FFQ, *MDS* Mediterranean Diet Score

The effects of these biomedical and lifestyle factors in the same genera were either smaller or even in the opposite direction in unaffected controls, as indicated by significant interactions. However, in the stratified analysis of the control groups alone, none of these were statistically significant.

In the diabetes cohort, current smokers exhibited a higher abundance of the genus *Bilophila* (Coef_cases_ = 1.31). Additionally, T2D patients with a higher BMI demonstrated reduced abundances of *Catabacter* (Coef_cases_ =  − 0.65). Furthermore, higher values of both diet scores were associated with an increased abundance of the genus *Mitsuokella* in the group of cases (HEI: Coef_cases_ = 1.15, MDS: Coef_cases_ = 1.21). This underscores the importance of diet and its impactful influence on the microbiome.

For the IBD-KC cohort, the following significant associations were found: IBD patients who are current smokers or exhibited a propensity for diarrhea-like stool types showed decreased abundances of *Oscillibacter* (Coef_cases_: Smoker status =  − 0.21; Bristol stool scale =  − 0.25). Additionally, female IBD patients show a higher abundance of *Oscillibacter* (Coef_cases_ = 0.17). Those with a higher frequency of diarrhea-like stool types additionally demonstrated reduced abundances of *Clostridium IV* (Coef_cases_ =  − 0.35) and *Subdoligranulum* (Coef_cases_ =  − 0.21). In return, current smokers further exhibited lower abundances of *Intestimonas* (Coef_cases_ =  − 0.37) and *Catabacter* (Coef_cases_ =  − 0.44). Lastly, higher calprotectin rates in IBD patients were linked to an increased abundance of *Flavonifractor* (Coef_cases_ = 0.18) and *Lactobacillus* (Coef_cases_ = 0.44).

## Discussion

The increasing prevalence of non-communicable diseases (NCDs) related to inflammation over the past few decades, particularly the nearly doubled incidence of type 2 diabetes (T2D, low-grade chronic inflammation) and inflammatory bowel disease (IBD, high-grade chronic inflammation) within the last 35 years, underscores the urgency of understanding the interplay between biomedical and lifestyle factors, disease prevalence, and timing of disease-onset. The opening remarks highlight the evident connection between changing biomedical and lifestyle factors and the increase in NCDs, establishing the context for an in-depth investigation into the diverse relationships uncovered in our primary findings.

Our analysis describes a relevant link between biomedical and lifestyle factors (anthropometric, lifestyle, metabolic, and disease-specific factors) and age-at-disease-onset, an important dimension for prevention strategies. For example, in diabetes, an earlier onset correlates with a higher risk for secondary complications and overall multimorbidity [33, 34]. However, associations with age-at-disease-onset are often overlooked. Across various NCDs, biomedical and lifestyle factors were related to both, disease prevalence and age-at-disease-onset, particularly in T2D and IBD. Higher Healthy Eating Index (HEI) scores contributed to a decreased hazard ratio for the age-at-disease-onset in IBD (HR = 0.8) and T2D (HR = 0.46), highlighting the importance of a high quality diet in disease prevention. Further exploration revealed that in IBD, greater microbiome diversity halved the hazard ratio, leading to a later disease onset. In T2D, triglyceride levels, a well-established marker of atherogenesis [35] and cardiovascular risk in T2D [36], demonstrated relevant contribution, while the microbiome exhibited fewer specific correlations. This suggests that certain biomedical and lifestyle factors, such as diet, play a key role in potentially delaying disease onset.

Comparing our findings with previous studies reveals consistent parallels in the impact of biomedical and lifestyle factors on the age of onset across various diseases, including Parkinson’s disease [37], cardiovascular disease [38], and dementia [39]. Our study, focused on inflammatory bowel disease (IBD) and diabetes, aligns with these trends. For example, sleep disturbance was associated with IBD, whereas recreational exercise was found to decrease the risk to develop IBD [40]. Another study highlighted the influence of diet on inflammatory activity and symptoms [41]. Regarding diabetes, multiple studies emphasize biomedical and lifestyle factors such as physical activity, smoking, diet, and body mass index (BMI) on disease risk [42, 43]. Lascar et al. describe the contribution of adiposity and unfavorable Western dietary choices to an earlier age-at-diabetes-onset [44], while the Mediterranean diet is linked to a significantly reduced T2D risk [45, 46]. Our findings align with these studies, particularly the role of high BMI in combination with an overall unfavorable dietary pattern as a driving force for diabetes onset. However, in IBD, diet and physical activity did not show significant associations after adjusting for sex and BMI. Interestingly, smoking showed significant associations with a delayed age-at-IBD-onset for UC patients, but not for CD patients. Similar observations have been reported in studies such as Berkowski et al. and Mahid et al. [47, 48].

Unexpectedly, our study identified a higher risk of developing T2D associated with high Mediterranean Diet Score (MDS). This finding may be explained by dietary recommendations for diabetes patients resembling a Mediterranean diet [49] and the limitation due to the case–control study design of no information on dietary habits before disease onset. Additionally, IBD showed an interesting association between high alcohol consumption and a lower risk of developing the disease, aligning with previous findings [50, 51]. This could be explained by IBD patients limiting alcohol intake due to medication interactions or to avoid triggering flare-ups, potentially influencing the observed association.

One limitation of our study is the case–control study design, which does not allow estimation of age-specific prevalences and limits interpretation to relative disease risks. In our case–control study, interpreting time-to-event analysis with age-at-disease-onset is also limited due to potential recruitment bias influenced by age-at-onset, possibly violating the assumption of independent censoring. While a cohort starting from birth would be ideal theoretically, the study’s design was deemed acceptable [52] considering the high age-at-onset for diabetes [53] and IBD [54] and data availability. The case–control design hinders absolute age-specific risk calculation due to case enrichment. Calculation of hazard ratios could be slightly biased: overestimated for cases and underestimated for controls. Despite these challenges, the study’s methodology is commonly employed [55], with the understanding that the results are applicable primarily to this case–control study population. Future research should transition to a cohort study design for enhanced generalizability. Another constraint of the family-based IBD-KC cohort and the case–control study design lies in the composition of the control group, which includes both first-degree relatives and genetically unrelated individuals. A previous study [56] identified differences in findings between IBD-unaffected family members and unrelated healthy population controls when examining the metagenome. Therefore, dividing the control group could yield slightly different findings and is advised for further investigations utilizing this cohort.

Given the relevance of the described biomedical and lifestyle factors in the timing of disease onset, these factors were further investigated on behalf of gut microbiome interaction. Uniformly, analysis of both T2D and IBD cohorts showed stronger interaction among diseased compared to healthy individuals. In IBD cases, notable findings comprise the association between male individuals, smokers, or those with diarrhea-like stool and lower abundances of the beneficial genus *Oscillibacter*. *Oscillibacter* is known for its beneficial impact on the gut microbiome [57], and therefore, the findings suggest potential medical interventions such as smoking cessation and dietary adjustments to enhance its abundance. Diarrhea-like stool types were also associated with reduced abundances of the strains *Clostridium IV* and *Subdoligranulum*, genera associated with potential probiotic benefits for human health and helpful in IBD treatment [9, 58, 59]. Smoking correlated with decreased abundances of *Intestinimonas*, a butyrate-producing genus. These findings suggest that improving microbiome diversity in terms of genera and discontinuing smoking could be potential preventative strategies for individuals at high risk. In T2D, smokers displayed elevated abundances of the pro-inflammatory bacterium *Bilophila* [60, 61], suggesting potential negative effects of smoking in diabetes patients mediated through alterations of this genus. Higher body mass index (BMI) was associated with decreased *Catabacter* abundances. We have also seen an association between increased *Halomonas* abundance and higher BMI. However, *Halomonas* did not reach initial thresholds in the controls group and has previously been identified as contamination, typically occurring in the water of buffers [62–64], which is why this association will not be further physiologically interpreted. Furthermore, a healthier diet, indicated by a high HEI and high MDS, was linked to higher abundances of *Mitsuokella*, a genus known for polysaccharide degradation and facilitation of protein digestion in the gut [65], which was just recently described to be positively associated with increased general health [66]. Notably, *Mitsuokella* was also identified as enriched in prediabetic patients [67]. Consequently, the characterization of *Mitsuokella* as beneficial or otherwise remains uncertain, necessitating further research. No significant associations could be identified for either of the healthy control groups. One plausible explanation for this absence could be the considerably lower variability observed in the control groups. For instance, when considering the Bristol stool scale, the control group exhibits minimal variability, with most individuals falling under the “normal” category. In contrast, the case group shows a more widespread distribution across the entire scale. A similar pattern is observed in variables like total and/or scaled energy intake, where the control group demonstrates greater homogeneity, while the cases exhibit a broader range of values. It is reasonable that individuals with IBD and T2D, being more cautious, might intentionally consume fewer calories to prevent flare-ups or due to dietary restrictions.

The impact of biomedical and lifestyle factors on the gut microbiome is a growing focus in scientific research. In IBD, stress can be linked to lower abundance of the beneficial genus *Lactobacillus* [68], and smoking increases the abundance of the genus *Prevotella* in both Crohn’s disease (CD) patients and healthy individuals [69]. We also found smoking to be influencing the abundance of genera, here, of *Oscillibacter*, *Intestinomas*, and *Catabacter*. The composition and richness of the gut microbiome are significantly linked to stool consistency. A positive association was observed between consistency and the *Bacteroidetes*:*Firmicutes* ratio, while individuals with the *Prevotella* enterotype tended to have looser stools [70]. These findings align with our study, where we found the abundances of *Oscillibacter*, *Clostridium IV*, and *Subdoligranulum* to be decreased in the gut microbiome of IBD patients with diarrhea-like stool types, emphasizing the notable influence of stool consistency on the gut microbiome composition. Diet plays a crucial role in both IBD and T2D, impacting the gut microbiome [71, 72]. In IBD, adherence to a Mediterranean diet has been found to benefit the gut microbiota by increasing fecal short-chain fatty acids (SCFA), likely influenced by bacteria from both Firmicutes and Bacteroidetes [73]. In a diet interventional study, Marlow et al. observed an increase in genera such as *Bacteroides*, *Clostridium IV*, and *Clostridium XIVa* when Crohn’s patients followed a 6-week Mediterranean diet [74]. We found *Clostridium IV* significantly decreased in the IBD-KC cohort in participants with a high MDS and HEI-EPIC score. However, no significant differences between the group of IBD patients and healthy controls were noticeable.

Similarly, in diabetes, the effect of nutrition-gut microbiome interactions is well recognized as influential in regard to weight and metabolic status [75]. For instance, a deficiency in SCFAs as the consequence of reduced levels of butyrate-producing gut bacteria from a low-fiber diet has been linked to impairments of central satiety sensing and glucose homeostasis [76]. In addition, another study demonstrated clinical improvement through altered gut microbiota under a fiber-rich diet, with increased abundance of genera such as *Lactobacillus*, *Akkermansia*, and *Ruminococcus*, and a decreased abundance of opportunistic genera like *Klebsiella*, *Megamonas*, and *Prevotella *[77]. A comparison between non-diabetics consuming a strict vegetarian, ovo-lacto-vegetarian, or omnivore diet further displayed compositional alterations of the gut microbiome with omnivores demonstrating an overrepresentation of *Succinivibrio*, which, in their study, was linked to an increased BMI, insulin resistance, and dyslipidemia [78]. Emphasizing the diet-gut microbiome interaction in T2D, our analysis also revealed significant associations between microbial genera, diet quality indices, and BMI among T2D cases. One aspect to be highlighted is the close resemblance of our findings regarding the Healthy Eating Index, which, similar to dietary patterns described in the literature, also reflects a sufficient intake of dietary fibers and limited consumption of animal products [31]. It is now also established that cigarette smoking exerts effects on the gut microbiome [79]. As already elaborated above, it is suggested that alterations display similarities to those observed with IBD [79]. However, there is currently no evidence for diabetes-specific interactions between smoking and gut microbiome composition. Overall, in the context of diabetes, studies on the interactions between biomedical and lifestyle factors and the gut microbiome are still limited and while our study parallels the previous literature, further studies are necessary for a full evaluation. We utilized only 16S data, focusing on the V1-V2 region, which may not capture the full complexity of the gut microbiome.

## Conclusions

Our study investigated the impact of anthropometric, metabolic, lifestyle, and disease-specific factors on the age-at-onset of non-communicable diseases, specifically IBD and T2D. We observed connections between these biomedical and lifestyle factors, including diet and disease-specific markers and a later disease onset, emphasizing the potential role of these factors in disease preventive strategies. We further identified effects of factors important for the timing of onset on the abundance of genera in the gut microbiome with smaller or even opposite effects in healthy compared to diseased individuals. This could indicate that the gut microbiome of T2D and IBD patients is more prone to negative influences of biomedical and lifestyle factors. To secure these findings, future research directions should involve diverse populations, consider transitioning to a cohort or prospective study design, and employ more comprehensive sequencing techniques to deepen our understanding of gut microbiota complexities. The study’s relevance lies in its contribution to understanding the complex relationships between biomedical and lifestyle factors, and chronic NCDs, not solely focusing on disease prevalence but also on timing and the role of the gut microbiome. Overall, our study lays further foundations for utilizing identified associations in personalized approaches and as preventive strategies and offers valuable insights for public health interventions amidst the rising global burden of non-communicable diseases.

## Supplementary Information

Additional file 1: Supplementary Table S1–S6, Figure S1-S2.

## Data Availability

All data are available upon request from the P2N biobank. Access token: P2N_7E22G; http://www.uksh.de/p2n/. P2N is a controlled-access human data repository subject to European data protection laws. Therefore, data access is subject to an application, ethics approval by the applicant’s ethics board and a data access agreement.

## Abbreviations

BW: Body weight
CD: Crohn’s disease
CI: Confidence interval
Coef: Coefficient
CS: Cross-sectional
ELISA: Enzyme linked immunosorbent assay
EPIC: European Investigation into Cancer and Nutrition
EtOH: Ethanol
FDR: False discovery rate
FFQ: Food frequency questionnaire
FoCus: Food Chain Plus Cohort
GLP-1: Glucagon-like peptide 1
HEI: Healthy Eating Index
HOMA-IR: Homeostatic model assessment for insulin resistance
HR: Hazard ratio
IBD: Inflammatory bowel disease
IBD-KC: Inflammatory Bowel Disease Kindred cohort
MaAsLin2: Microbiome Multivariable Associations with Linear Models
MDS: Mediterranean Diet Score
NCD: Non-communicable disease
OR: Odds ratio
SCFA: Short-chain fatty acid
T2D: Type 2 diabetes
UC: Ulcerative colitis
UKSH: University Medical Center Schleswig–Holstein
