# Supplementary material for: Metabolic and lifestyle factors accelerate disease onset and alter gut microbiome in inflammatory non-communicable diseases
Source: BMC Med. 2024 Oct 24;22:493. doi: 10.1186/s12916-024-03709-0 (PMC11515311; doi:10.1186/s12916-024-03709-0)
Supplement: Supplementary file 1 — Additional file 1: Supplementary Table S1–S6, Figure S1-S2. [file 12916_2024_3709_MOESM1_ESM.pdf]

Additional file

Table of contents

Material and Methods 2
Health status 2
Sleep duration, physical activity, and TV watching 2
Gut microbiota 2
Serum metabolite profiles 2
Data pre-processing 3
Details to Statistical analysis 3
Calculation of diet scores 3
Healthy Eating Index 3
Mediterranean Diet Score 4
Addition to the exploratory analysis of phenotype, lifestyle, microbial diversity and NCDs 6
Additional characterization of disease-specific factors for the T2D and IBD study populations 11
Beta diversity analysis for type 2 diabetes and inflammatory bowel disease 11
Addition to the association between biomedical and lifestyle factors and age-at-disease-onset 12
References 13

Material and Methods

Health status

A questionnaire for nutrition medical studies provided by the popgen biobank (v.1.1) was used to obtain self-reported disease status by structured questions regarding metabolic inflammatory diseases. Disease diagnoses were considered together with other relevant information, including the age-at-disease-onset and subclassification.

Sleep duration, physical activity, and TV watching

Information on the duration of sleep, physical activity, and TV watching were also collected through the EPIC-FFQ. Hours of sleep were given separately for daytime and nighttime (for the IBD-KC cohort additionally separated into summer and winter season, however a mean of the whole year was computed) and added up to define the total hours of sleep in a whole 24 hour day cycle. Information on physical activity were provided as daily mean of the whole year or separate summer and winter season based on if it is most likely performed in- or outdoor. For the latter, mean values were formed. Afterwards, moderate activities were summed as “everyday activity” including the hours per week spent doing housework, crafting, gardening, or walking. In return, “sports activity” was defined as the sum of hours per week cycling and performing other sports. The average duration of TV watching per week was also collected.

Gut microbiota

Fecal samples from study participants of both cohorts were collected at home using standard stool collection tubes and subsequently mailed to the study center. Samples were stored at −80°C until further processed by the Institute of Clinical Molecular Biology at Kiel University, Germany. DNA extraction from approximately 200 mg of fecal material was conducted using QIAamp DNA stool mini-kits, with automation on the QIAcube (QIAGEN). The subsequent steps involved 16S rRNA gene library preparation and sequencing, following a previously described protocol.[1,2] Specifically, the V1-V2 region of the 16S rRNA gene was sequenced on the MiSeq platform, employing v3 chemistry for 2 × 300 bp paired-end reads (Illumina Inc., San Diego, CA, United States). Data processing utilized the DADA2 version 1.10 workflow designed for large datasets (https://benjjneb.github.io/dada2/bigdata.html), resulting in Amplicon Sequence Variant (ASV) abundance tables. Different sequencing runs were managed separately (Github: @mruehlemann) for a V1-V2-adjusted workflow and were consolidated into a single abundance table per dataset only after final chimera filtering. ASVs underwent taxonomic annotation using the Bayesian classifier provided by DADA2 and the Ribosomal Database Project (RDP) version 16 release. All subsequent microbiota analyses were conducted at the genus level.

Serum metabolite profiles

Serum metabolomics profiling was performed from serum samples by the Department of Food Technology at Kiel University. In brief, samples were gently thawed and 1:1000 diluted with methanol and water (50:50, v/v) and quality controls were prepared following [3]. Data was acquired using the high performance liquid chromatography (1260 Infinity System, Agilent, Waldbronn, Germany) which was linked to an extreme high-resolution Fourier transform ion cyclotron resonance mass spectrometry (7T, SolariXR, Bruker, Bremen, Germany). Mass spectra were obtained by electrospray ionization in both positive and negative ionization mode and a mass range of 65-1500 Da. More details can be found in Seoudy et al..[4] Pooled QC samples were injected at the start and end of each batch. Data was processed in the MetaboScape 2021b software (Bruker, Bremen, Germany). Most probable chemical formulas were assigned based on accurate measured masses, isotopic patterns and the seven golden rules[5], whereas compound names were matched to the Human Metabolome Database v.5.0.[6] Signal correction, peak filtering and imputation of missing values were conducted using R package “statTarget” v.1.24.0.[7] Signal correction was applied using QC-based random forest. Peak filtering was conducted by only including compounds that were detected in at least 80% of samples. Imputation of missing values was performed using the k Nearest Neighbor method. Datasets were merged to one final data set. Three metabolite clusters were formed among subjects using the k-means clustering method (R packages “cluster”, v.2.1.4). Optimal number of clusters was determined using the silhouette-method.

Data pre-processing

Subjects with missing values (NA) across all variables were excluded. Individuals without assigned sex were also excluded, resulting in the exclusion of 58 subjects in the IBD-KC, and 4 in the Focus cohort. Additionally, in the IBD-KC cohort, subjects with a BMI exceeding 35 kg/m2 were excluded, leading to the removal of 44 subjects. Missing values for biomedical and lifestyle factors were imputed: Age (1 individual in IBD-KC; 3 in FoCus-CS; 1 in FoCus-T2D), height (19 individuals in IBD-KC) weight (31 individuals in IBD-KC), calprotectin (45 individuals in IBD-KC), waist circumference (10 individuals in FoCus-CS), hip circumference (14 individuals in FoCus-CS), glucose (3 individuals in FoCus-CS; 2 in FoCus-T2D), insulin (5 individuals in FoCus-CS; 3 in FoCus-T2D), CRP (2 individuals in FoCus-CS; 1 in FoCus-T2D), IL-6 (2 in FoCus-CS), triglycerides (2 individuals in FoCus-CS; 1 in FoCus-T2D), and GLP-1 (116 individuals in FoCus-T2D) were imputed using group-specific (cases/controls in IBD-KC and FoCus-T2D) and sex-specific (male/female in FoCus-CS) median values. For smoking, missing values were replaced with 0 by default, indicating no current smoking habits (5 individuals in IBD-KC, 27 individuals in FoCus-CS, 13 individuals in FoCus-T2D). Missing values of chronic disease diagnoses were also replaced with 0 by default, indicating the absence of disease (T2D: 9; arterial hypertension: 11; hyperlipidemia: 25; chronic heart failure: 20; coronary artery disease: 36; rheumatoid arthritis: 39; IBD: 17; respiratory disease (asthma bronchiale/chronic bronchitis): 43); skin disease (psoriasis): 461. The estimated prevalence for psoriasis in Germany is around 2% [8], hence it is reasonable to assume that many missing values indicate no disease.

Details to Statistical analysis

Exploratory analysis of phenotype and biomedical and lifestyle factors with NCDs

Adjustments made for sex and BMI class, were done according to WHO thresholds: underweight (UW) < 18.5 kg/m2, normal weight (NW) 18.5 - 24.9 kg/m2, overweight (OW) 25 - 29.9 kg/m2, and obesity I° (OI) 30 - 34.9 kg/m2, II° (OII) 35 - 39.9 kg/m2 and III° (OIII) ≥ 40 kg/m2.[9]

Association between biomedical and lifestyle factors and age-at-disease-onset

Continuous biomedical and lifestyle factors were categorized into either terciles or deciles for analysis and graphical visualization because those factors don’t follow a log- link distribution. As described above, BMI was categorized into BMI classes. For IBD, obesity classes were grouped together since the BMI threshold was set at BMI < 35 kg/m2. Diet scores (MDS and HEI) were grouped into three categories: "high," "moderate,", and "low", ensuring an equal distribution of participants in each group. Decile transformation was applied to total energy quantity, scaled energy quantity, alcohol, activity (everyday, sports, TV watching), sleep, Shannon index, Chao1 index, and calprotectin. For a more intuitive representation, the Bristol Stool Scale (only in IBD-KC cohort) was transformed into a numerical scale: 3+4 mapped to 1 (normal stool), 2 mapped to 2 (mild constipation), 1 mapped to 3 (severe constipation), 5 mapped to 5 (missing fiber), 6 mapped to 5 (mild diarrhea), and 7 mapped to 6 (severe diarrhea).

Role of biomedical and lifestyle-gut microbiota interactions in diabetes and IBD

We used the R package Maaslin2 (Microbiome Multivariable Association with Linear Models)[10], version (1.16.0) with the following settings: analysis method: CPLM (Compound Poisson Linear Model), normalization_ TSS (total sum scaling), transformation: log link (default), minimal abundance: 10 (counts), minimum prevalence: 0.1 (percent of samples for which a genera is detected at minimum abundance, default).

Calculation of diet scores

Healthy Eating Index

The Healthy Eating Index indicates how closely the individual’s diet conforms to defined dietary recommendations, whereby the here used adapted form (HEI-EPIC) is in line with the “German food

pyramid recommendations” published by the German Federal Agency for Agriculture and Food.[11] We computed the index by adapting the calculation from von Rüsten et al. who specifically adjusted each step to fit the EPIC-FFQ data.[12] Index values range from 0-100 with higher values reflecting a better conformation to guidelines. Nine food groups that are split into three scoring-categories are considered: (1) adequation (drinks, vegetables, and fruits), (2) moderation (sweets/fatty snacks) and (3) in-betweens (grains/grain products/potatoes, milk/dairy produce, meat/processed meat/fish/eggs, fats and oils). Respectively, scores are given with either increasing or decreasing consumption or as a combination of both. In the latter case, if participants whose consumption of the target category did not exceed the recommendation, scoring was performed according to adequation. If the recommendation was exceeded, scoring was performed according to moderation. This considers that, although a moderate amount of these foods is recommended, excessive consumption, however, makes a significant contribution to the daily energy intake.[12] Details on the HEI-EPIC composition and calculation are provided in **Supplementary Table S1**.

Table S1: Composition and calculation of the HEI-EPIC

**Food group**	**Maximum score**	**Recommendation, servings/day[a]**	**Included foods from the EPIC-FFQ v.1**
Adequation = daily intake/recommendation × 10			
Water	10	≥ 6	Water
Vegetables	20	≥ 3	All vegetables, ½ juices
Fruits	20	≥ 2	Fruits, mixed fruits, ½ juices
Moderation = recommendation/daily intake × 10			
Sweets and salty snacks	10	≤ 1	Sweets, cake, snacks
In-betweens (> recommendation) = recommendation/daily intake × 10<br>*In-betweens (≤ recommendation) = daily intake/recommendation × 10*			
Grains, grain product and potatoes	10	4	Potatoes, crispbread, bread, noodles, pasta, rice
Milk and dairy products	10	3	Milk, yogurt, cheese
Meat, meat products, fish and eggs	10	1	All meat, fish, eggs
Fats and oils	10	2	All fats
**HEI-EPIC (0-100)**	**= Sum of all scores**		

[a] recommended serving sizes according to AID GERMANY: water = 280 ml; juice = 100 ml; vegetables = 140 g; fruits = 125 g; grains/bread = 85 g (m), 70 g (f); potatoes/pasta/noodles/rice (cooked) = 250 g (m), 200 g (f); milk = 250 ml; yogurt = 150 g; cheese = 30 g; meat = 30 g; fish = 150 g; eggs = 2 ≙ 16 g, fats/oils = 20 g (m), 18 g (f); sweets/salty snacks = 270 kcal (m), 220 kcal (f). Abbreviations: HEI-EPIC, Healthy Eating Index specifically modified to fit the EPIC-FFQ data; m, male, f, female; kcal, kilocalorie. Calculation based on Rüsten et al..[12]

Mediterranean Diet Score

The Mediterranean Diet Score (MDS) has originally been established in southern Europe [13] to indicate how much the individual’s diet adheres to a Mediterranean dietary pattern, which has been recognized for its anti-inflammatory, anti-atherosclerotic and overall life-prolonging properties [14]. Ever since, the evaluation of this index has been used in other parts of the continent, whereby its constitution has slightly been adapted to suit non-Mediterranean populations [15]. The calculation includes nine food groups whose consumption contributes significantly to the described health benefits of the Mediterranean Diet: (1) vegetables, (2) legumes, (3) fruit and nuts, (4) cereals, (5) fish and fish products are considered beneficial food groups. In return, (6) milk and dairy, as well as (7) meat and meat products are considered obstructive food groups. Sex-specific medians of the daily intake of these food groups are formed, which are then compared with the individual intake. If the daily intake of the beneficial food groups is equal to or greater than the median, one point is awarded. For the obstructive food groups, one point is awarded if the daily intake is below the median. Two further groups are awarded a point:

(8) a moderate alcohol intake of 10-50 g ethanol/day for men and 5-25 g ethanol/day for women and (9) a high fatty acid ratio (PUFAs + PUFAs / SFAs ≥ median), resulting in a scoring range of 0-9 [15].

Details on the MDS composition and calculation are provided in **Supplementary Table S2**.

Table S2: Composition and calculation of the MDS

**Food group**	**Maximum score**	**Calculation**	**Included foods from the EPIC-FFQ v.1**
Beneficial food groups			
Vegetables	1	1: Daily intake ≥ sex-specific median<br>0: Daily intake < sex-specific median	Green leafy vegetables, fruit vegetables, root vegetables, cabbage, mushrooms, pod vegetables, onions, stalk vegetables, mixed salad, mixed vegetables
Legumes	1	1: Daily intake ≥ sex-specific median<br>0: Daily intake < sex-specific median	Legumes
Fruits and nuts	1	1: Daily intake ≥ sex-specific median<br>0: Daily intake < sex-specific median	Fruits, nuts and seeds, mixed fruits
Whole grain cereal	1	1: Daily intake ≥ sex-specific median<br>0: Daily intake < sex-specific median	Flour, grain flakes, starch, bread, crispbread, rusk
Fish	1	1: Daily intake ≥ sex-specific median<br>0: Daily intake < sex-specific median	Fish, crustaceans, fish products
Obstructive food groups			
Dairy	1	1: Daily intake < sex-specific median<br>0: Daily intake ≥ sex-specific median	Milk, milk drinks, yogurt, quark, cheese, cream desserts, pudding, cream, sour cream, coffee cream, coffee whitener
Meat	1	1: Daily intake < sex-specific median<br>0: Daily intake ≥ sex-specific median	Red meats (beef, veal/calf, pork)
Specific consideration			
Fats	1	(PUFAs+MUFAs)/SFAs	PUFAs, MUFAs, SFAs
Alcohol	1	1: Within moderate consumption rate<br>- m: 10-50 g/day<br>- f: 5-25 g/day<br>0: Outside of moderate consumption rate	Sum ethanol [g/day]
**MDS**	**= Sum of all scores**		

Abbreviations: MDS, Mediterranean Diet Score; PUFA, Poly-unsatturated fatty acid; MUFA, Mono-unsatturated fatty acid; SFA, Saturated fatty acid; m, male, f, female; kcal, kilocalorie. Calculation based on Trichopoulou et al..[15]

Addition to the exploratory analysis of phenotype, lifestyle, microbial diversity and NCDs

Table S3: Exploratory analysis of phenotype, lifestyle, microbial diversity and NCDs using unadjusted logistic regression models.

**Biomedical and lifestyle factors**	**Odds ratio [95% CI]**	***p***
Obesity		
Waist cir. [cm]	1.2 [1.17-1.23]	$< 10^{-5}$
Hip cir. [cm]	1.37 [1.31-1.42]	$< 10^{-5}$
WHR	64088.6 [9623.65-426797.32]	$< 10^{-5}$
Glucose [mg/dl]	1.02 [1.01-1.03]	$< 10^{-5}$
Insulin [µU/l]	1.08 [1.06-1.1]	$< 10^{-5}$
HOMA-IR	1.2 [1.14-1.26]	$< 10^{-5}$
CRP [pg/l]	1.05 [1.03-1.08]	6.94×10^{-5}
IL-6 [pg/l]	1.03 [1.01-1.05]	6.85×10^{-3}
Triglycerides [mmol/l]	1.01 [1.01-1.01]	$< 10^{-5}$
Scaled energy intake [kcal/kg BW/d]	0.87 [0.85-0.89]	$< 10^{-5}$
MDS [0-9]	0.85 [0.77-0.94]	1.04×10^{-3}
HEI-EPIC [0-100]	0.98 [0.97-1]	1.91×10^{-2}
Fats [EN%]	0.97 [0.94-0.99]	1.36×10^{-2}
Proteins [EN%]	1.21 [1.12-1.3]	$< 10^{-5}$
Alcohol [g EtOH/d]	0.96 [0.92-1]	3.34×10^{-2}
Sports [h/week]	0.9 [0.86-0.94]	$< 10^{-5}$
TV watching [h/week]	1.02 [1.01-1.03]	1.94×10^{-5}
Smoke intensity [n cig./d]	1.02 [1.01-1.04]	5.05×10^{-4}
Type 2 Diabetes		
Waist cir. [cm]	1.06 [1.05-1.08]	$< 10^{-5}$
Hip cir. [cm]	1.05 [1.03-1.08]	$< 10^{-5}$
WHR	5019.1 [388.85-64784.45]	$< 10^{-5}$
Glucose [mg/dl]	1.05 [1.03-1.06]	$< 10^{-5}$
Insulin [µU/l]	1.04 [1.02-1.05]	$< 10^{-5}$
HOMA-IR	1.14 [1.09-1.19]	$< 10^{-5}$
Triglycerides [mmol/l]	1 [1-1.01]	2.39×10^{-5}
Scaled energy intake [kcal/kg BW/d]	0.93 [0.9-0.96]	2.9×10^{-5}
Carbohydrates [EN%]	0.95 [0.91-0.99]	1.62×10^{-2}
Fats [EN%]	1.05 [1-1.11]	3.5×10^{-2}
Proteins [EN%]	1.16 [1.04-1.31]	1.04×10^{-2}
Sports [h/week]	0.89 [0.82-0.96]	3.75×10^{-3}
TV watching [h/week]	1.02 [1.01-1.03]	6.4×10^{-4}
Sleep [h/24h]	1.24 [1-1.53]	4.85×10^{-2}
Smoke intensity [n cig./d]	1.06 [1.04-1.08]	$< 10^{-5}$
Arterial hypertension		
Waist cir. [cm]	1.06 [1.05-1.07]	$< 10^{-5}$
Hip cir. [cm]	1.06 [1.04-1.07]	$< 10^{-5}$
WHR	2646.71 [631.69-11089.45]	$< 10^{-5}$
Glucose [mg/dl]	1.02 [1.01-1.02]	$< 10^{-5}$
Insulin [µU/l]	1.04 [1.03-1.06]	$< 10^{-5}$
HOMA-IR	1.11 [1.06-1.15]	$< 10^{-5}$
CRP [pg/l]	1.05 [1.02-1.07]	3.5×10^{-4}
IL-6 [pg/l]	1.03 [1-1.05]	1.68×10^{-2}
Triglycerides [mmol/l]	1.01 [1-1.01]	$< 10^{-5}$
Scaled energy intake [kcal/kg BW/d]	0.97 [0.95-0.98]	$< 10^{-5}$
Carbohydrates [EN%]	0.97 [0.95-0.99]	3.66×10^{-3}
Proteins [EN%]	1.08 [1.02-1.14]	1.04×10^{-2}
Alcohol [g EtOH/d]	1.04 [1.02-1.07]	1.9×10^{-3}
Everyday activity [h/week]	1.02 [1.01-1.02]	2.92×10^{-4}
Sports [h/week]	0.95 [0.93-0.98]	1.09×10^{-3}

TV watching [h/week]	1.02 [1.01-1.03]	1.94×10^{-5}
Current smoker [no/yes]	0.69 [0.49-0.97]	3.28×10^{-2}
Smoke intensity [n cig./d]	1.02 [1.01-1.03]	2.1×10^{-4}
Hyperlipidemia		
Waist cir. [cm]	1.03 [1.02-1.03]	$< 10^{-5}$
WHR	139.91 [36.3-539.3]	$< 10^{-5}$
Glucose [mg/dl]	1.01 [1-1.01]	1.88×10^{-3}
Insulin [µU/l]	1.01 [1-1.02]	1.34×10^{-2}
HOMA-IR	1.04 [1.01-1.07]	4.67×10^{-3}
Triglycerides [mmol/l]	1.01 [1.01-1.01]	$< 10^{-5}$
MDS [0-9]	1.21 [1.11-1.32]	$< 10^{-5}$
Everyday activity [h/week]	1.01 [1-1.02]	1.22×10^{-2}
TV watching [h/week]	1.01 [1-1.02]	2.15×10^{-2}
Current smoker [no/yes]	0.65 [0.45-0.94]	2.14×10^{-2}
Smoke intensity [n cig./d]	1.03 [1.02-1.04]	$< 10^{-5}$
Chronic heart failure		
CRP [pg/l]	1.06 [1.03-1.09]	2.82×10^{-4}
Sports [h/week]	0.88 [0.78-0.99]	3.85×10^{-2}
Shannon index	0.4 [0.18-0.89]	2.53×10^{-2}
Coronary artery disease		
Waist cir. [cm]	1.03 [1.01-1.05]	9.97×10^{-4}
WHR	377.81 [26.36-5414.22]	1.25×10^{-5}
Insulin [µU/l]	1.01 [1-1.02]	0.07
Triglycerides [mmol/l]	1 [1-1.01]	3.78×10^{-2}
MDS [0-9]	1.2 [1-1.44]	4.6×10^{-2}
TV watching [h/week]	1.01 [1-1.03]	3.43×10^{-2}
Sleep [h/24h]	1.3 [1.03-1.64]	2.48×10^{-2}
Current smoker [no/yes]	0.19 [0.05-0.8]	2.31×10^{-2}
Smoke intensity [n cig./d]	1.04 [1.02-1.06]	1.86×10^{-4}
Rheumatoid arthritis		
WHR	6.23 [0.77-50.16]	0.09
CRP [pg/l]	1.06 [1.03-1.08]	1.70×10^{-5}
IL-6 [pg/l]	1.03 [1.01-1.05]	5.88×10^{-3}
Total energy intake [kcal/d]	1 [1-1]	0.06
Carbohydrates [EN%]	1.04 [1.01-1.08]	2.41×10^{-2}
Everyday activity [h/week]	1.02 [1.01-1.03]	6.27×10^{-3}
Psoriasis		
Alcohol [g EtOH/d]	1.05 [0.99-1.12]	0.096
Smoke intensity [n cig./d]	1.04 [1.02-1.07]	1.83×10^{-3}
Inflammatory bowel disease		
Hip cir. [cm]	0.97 [0.94-0.99]	1.40×10^{-2}
WHR	9.04 [0.92-89.21]	0.06
CRP [pg/l]	1.06 [1.03-1.08]	3.23×10^{-5}
Triglycerides [mmol/l]	1 [1-1]	3.63×10^{-2}
Proteins [EN%]	1.12 [1-1.25]	4.53×10^{-2}
Sports [h/week]	0.93 [0.87-0.99]	2.81×10^{-2}
Smoke intensity [n cig./d]	1.02 [1-1.04]	0.05
Asthma bronchiale		
Waist cir. [cm]	1.02 [1-1.03]	3.13×10^{-2}
Hip cir. [cm]	1.04 [1.02-1.06]	7.77×10^{-5}
Scaled energy intake [kcal/kg BW/d]	0.98 [0.96-1]	0.09
Proteins [EN%]	1.11 [1-1.24]	0.06
Alcohol [g EtOH/d]	0.92 [0.86-1]	3.93×10^{-2}
Shannon index	0.39 [0.23-0.66]	5.57×10^{-4}
Chao1 index	0.98 [0.96-1]	0.07
Chronic bronchitis		

Waist cir. [cm]	1.04 [1.03-1.06]	$< 10^{-5}$
Hip cir. [cm]	1.06 [1.04-1.09]	$< 10^{-5}$
WHR	34.49 [2-593.77]	1.47×10^{-2}
Glucose [mg/dl]	1.01 [1-1.02]	3.53×10^{-3}
HOMA-IR	1.04 [1-1.07]	3.64×10^{-2}
CRP [pg/l]	1.04 [1.01-1.07]	2.24×10^{-2}
IL-6 [pg/l]	1.02 [1-1.03]	9.19×10^{-3}
Scaled energy intake [kcal/kg BW/d]	0.96 [0.93-0.99]	2.31×10^{-2}
Sports [h/week]	0.86 [0.78-0.95]	2.66×10^{-3}
Sleep [h/24h]	0.75 [0.6-0.96]	1.96×10^{-2}
Smoke intensity [n cig./d]	1.04 [1.01-1.06]	1.48×10^{-3}

Abbreviations: BMI. Body Mass Index; WHR. waist-to-hip ratio; HOMA-IR. Homeostatic model assessment of insulin resistance; CRP. C-reactive protein; IL. interleukin; MDS. Mediterranean Diet Score; HEI-EPIC. Healthy Eating Index derived from EPIC-FFQ data; BW. body weight.

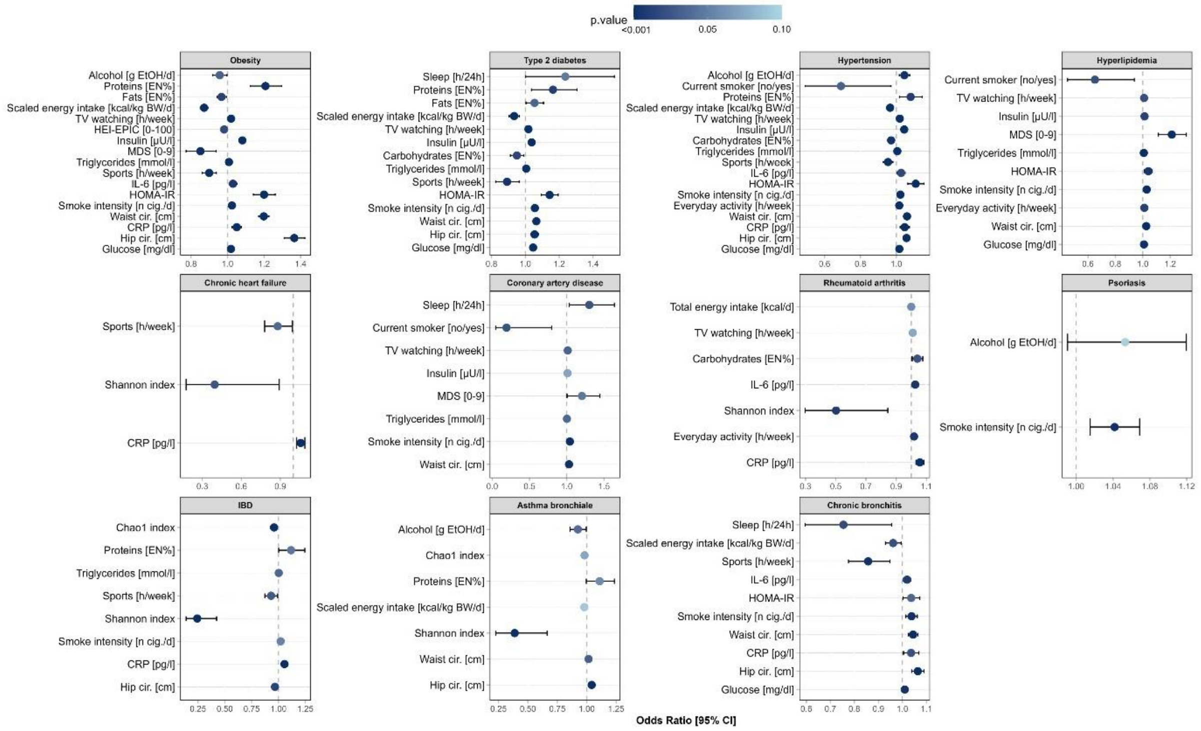

Figure S1: Exploratory analysis of phenotype and medical and lifestyle factors in association with common NCDs using unadjusted logistic regression models

Presented are results of the exploratory analysis of 11 NCDs in relation to selected biomedical and lifestyle factors using unadjusted logistic regression models (disease presence/absence). Odds Ratios (OR) estimated from factors with $p < 0.1$ (dot color indicating p-value). Due to very high ORs. associations with waist-to-hip ratio are not displayed. Abbreviations: NCD. non-communicable disease; MDS. Mediterranean Diet Score; HEI-EPIC. Healthy Eating Index determined from EPIC-FFQ; HOMA-IR. Homeostatic Model Assessment of Insulin Resistance; CRP. C-reactive protein; IL. interleukin; IBD. inflammatory bowel disease; BMI. Body Mass Index.

Table S4: Exploratory analysis of phenotype. lifestyle. microbial diversity and NCDs using adjusted logistic regression models.

Biomedical and lifestyle factors	Odds ratio [95% CI]	*p*
Obesity		
Waist cir. [cm]	1.28 [1.24-1.32]	$< 10^{-5}$
Hip cir. [cm]	1.38 [1.32-1.44]	$< 10^{-5}$
WHR	1.91×10^{8} [1.05×10^{7}-3.46×10^{9}]	$< 10^{-5}$
Glucose [mg/dl]	1.02 [1.01-1.03]	$< 10^{-5}$
Insulin [µU/l]	1.08 [1.06-1.1]	$< 10^{-5}$
HOMA-IR	1.2 [1.14-1.26]	$< 10^{-5}$
CRP [pg/l]	1.05 [1.02-1.08]	6.98×10^{-5}
IL-6 [pg/l]	1.03 [1.01-1.05]	8.75×10^{-3}
Triglycerides [mmol/l]	1.007 [1.005-1.009]	$< 10^{-5}$
Scaled energy intake [kcal/kg BW/d]	0.87 [0.85-0.89]	$< 10^{-5}$
MDS [0-9]	0.84 [0.76-0.93]	4.09×10^{-4}
HEI-EPIC [0-100]	0.98 [0.96-1]	1.87×10^{-2}
Carbohydrates [EN%]	1.03 [1-1.06]	2.99×10^{-2}
Fats [EN%]	0.96 [0.93-0.99]	5.08×10^{-3}
Proteins [EN%]	1.21 [1.12-1.3]	$< 10^{-5}$
Alcohol [g EtOH/d]	0.95 [0.91-0.99]	1.31×10^{-2}
Sports [h/week]	0.9 [0.86-0.94]	$< 10^{-5}$
TV watching [h/week]	1.02 [1.01-1.03]	3.97×10^{-5}
Sleep [h/24h]	0.9 [0.79-1.02]	0.09
Smoke intensity [n cig./d]	1.02 [1.01-1.03]	1.99×10^{-3}
Type 2 Diabetes		
BMI [kg/m^{2}]	1.06 [1-1.12]	2.4×10^{-2}
Waist cir. [cm]	1.06 [1.03-1.09]	$< 10^{-5}$
Hip cir. [cm]	1.04 [1.01-1.08]	4.42×10^{-2}
WHR	5.95×10^{2} [2.17×10^{1}-1.63×10^{4}]	1.55×10^{-4}
Glucose [mg/dl]	1.04 [1.03-1.05]	$< 10^{-5}$
Insulin [µU/l]	1.02 [1-1.04]	3.42×10^{-3}
HOMA-IR	1.11 [1.06-1.16]	$< 10^{-5}$
Triglycerides [mmol/l]	1.004 [1.001-1.006]	1.61×10^{-3}
Scaled energy intake [kcal/kg BW/d]	0.95 [0.92-0.98]	4.98×10^{-3}
Fats [EN%]	1.05 [1. 1.11]	0.07
Proteins [EN%]	1.13 [0.99-1.28]	0.06
Sports [h/week]	0.93 [0.86-1]	0.05
TV watching [h/week]	1.01 [1-1.02]	4.12×10^{-2}
Smoke intensity [n cig./d]	1.05 [1.03-1.07]	2.28×10^{-5}
Arterial hypertension		
BMI [kg/m^{2}]	1.13 [1.08-1.18]	$< 10^{-5}$
Waist cir. [cm]	1.05 [1.03-1.07]	$< 10^{-5}$
Hip cir. [cm]	1.03 [1.01-1.05]	6.68×10^{-4}
WHR	229.54 [28.3-1.86×10^{3}]	$< 10^{-5}$
Insulin [µU/l]	1.02 [1.01-1.03]	1.02×10^{-3}
HOMA-IR	1.04 [1-1.08]	$4.04 \times 10\text{-}^{-2}$
CRP [pg/l]	1.04 [1.01-1.07]	2.61×10^{-3}
Triglycerides [mmol/l]	1.003 [1.001-1.005]	1.28×10^{-3}
Scaled energy intake [kcal/kg BW/d]	0.98 [0.97-0.99]	5.91×10^{-3}
HEI-EPIC [0-100]	0.99 [0.98-1.01]	0.05
Alcohol [g EtOH/d]	1.04 [1.01-1.07]	$2..66 \times 10^{-2}$
Sports [h/week]	0.97 [0.94-1]	4.64×10^{-2}
Hyperlipidemia		
WHR	11.24 [1.59-79.42]	1.53×10^{-2}
Triglycerides [mmol/l]	1.01 [1.01-1.01]	$< 10^{-5}$
MDS [0-9]	1.17 [1.07-1.28]	4.1×10^{-4}

Smoke intensity [n cig./d]	1.02 [1.01-1.03]	9.15×10^{-3}
Chronic heart failure		
CRP [pg/l]	1.06 [1.02-1.1]	5.12×10^{-4}
Shannon index	0.26 [0.1-0.66]	4.11×10^{-4}
Coronary artery disease		
Triglycerides [mmol/l]	1.003 [0.9995-1.006]	0.09
Rheumatoid arthritis		
CRP [pg/l]	1.06 [1.03-1.09]	1.64×10^{-5}
IL-6 [pg/l]	1.02 [1-1.04]	1.41×10^{-2}
Carbohydrates [EN%]	1.05 [1.01-1.09]	8.16×10^{-3}
Proteins [EN%]	0.91 [0.81-1.02]	0.09
Alcohol [g EtOH/d]	0.94 [0.88-1]	0.06
Shannon index	0.41 [0.24-0.71]	1.34×10^{-3}
Psoriasis		
Waist cir. [cm]	0.95 [0.92-0.98]	2.89×10^{-3}
Smoke intensity [n cig./d]	1.04 [1.01-1.07]	1.29×10^{-2}
Inflammatory bowel disease		
Hip cir. [cm]	0.95 [0.92-0.98]	3.35×10^{-3}
WHR	133.11 [6.09-1.86 x 10^3]	1.89×10^{-3}
CRP [pg/l]	1.06 [1.03-1.09]	3.05×10^{-5}
Triglycerides [mmol/l]	1.003 [1.0004-1.005]	2.1×10^{-2}
Proteins [EN%]	1.13 [1.01-1.26]	3.61×10^{-2}
Sports [h/week]	0.93 [0.87-0.99]	2.11×10^{-2}
Smoke intensity [n cig./d]	1.02 [1-1.04]	3.60×10^{-2}
Shannon index	0.25 [0.14-0.44]	$< 10^{-5}$
Chao1 index	0.96 [0.94-0.98]	1.06 x 10-^{4}
Asthma bronchiale		
BMI [kg/m^2]	1.06 [1.01-1.11]	1.35×10^{-2}
Waist cir. [cm]	1.03 [1.01-1.05]	9.8×10^{-4}
Hip cir. [cm]	1.04 [1.01-1.07]	4.37×10^{-3}
MDS [0-9]	1.15 [0.99-1.34]	0.08
Proteins [EN%]	1.12 [1-1.25]	0.06
Shannon index	0.41 [0.24-0.71]	1.87×10^{-3}
Chao1 index	0.98 [0.96-1]	0.08
Chronic bronchitis		
Waist cir. [cm]	1.03 [1-1.06]	0.06
Glucose [mg/dl]	1.01 [1-1.02]	0.07
CRP [pg/l]	1.03 [0.99-1.07]	0.08
Sports [h/week]	0.9 [0.81-0.99]	3.23×10^{-2}
Sleep [h/24h]	0.8 [0.63-1.01]	0.06
Current smoker [no/yes]	2 [0.96-4.18]	0.07
Smoke intensity [n cig./d]	1.04 [1.01-1.07]	4.02×10^{-3}
Chao1 index	0.98 [0.96-1.01]	0.09

Logistic regression is adjusted for age, sex (outcome obesity) and additionally obesity status (all other NCDs). Abbreviations: BMI. Body Mass Index; WHR. waist-to-hip ratio; HOMA-IR. Homeostatic model assessment of insulin resistance; CRP. C-reactive protein; IL. interleukin; MDS. Mediterranean Diet Score; HEI-EPIC. Healthy Eating Index derived from EPIC-FFQ data; BW. body weight.

Additional characterization of disease-specific factors for the T2D and IBD study populations

Table S5A: Characterization of disease-specific factors for T2D

Disease-specific factors	Cases	Controls	p^a
Subjects. n	197	317	-
Glucose. mg/dl	124 [107. 162]	90 [86. 94]	$< 10^{-5}$
Insulin. µU/l	23.6 [13.6. 44.2]	5.7 [4.32. 7]	$< 10^{-5}$
HOMA-IR	7.22 [3.96. 13.45]	1.24 [0.96. 1.58]	$< 10^{-5}$
Triglycerides. mg/dl	157 [119. 238]	73 [59. 95]	$< 10^{-5}$
GLP-1. pg/ml	10.17 [4.44. 15.72]	5.02 [4.18. 5.82]	$< 10^{-5}$
Metabolite clusters. prevalence (95% CI)			0.38
- 1	0.38 (0.31. 0.45)	0.37 (0.32. 0.43)	
- 2	0.51 (0.44. 0.58)	0.47 (0.42. 0.53)	
- 3	0.11 (0.07. 0.16)	0.15 (0.12. 0.20)	

Data is displayed as median [Interquartile Range]. [a] Wilcoxon-test for numerical variables; Chi^2test for categorical variables. Abbreviations: T2D. Type 2 diabetes; HOMA-IR. Homeostatic model assessment for insulin resistance; GLP-1. Glucagon-like peptide 1.

Table S5B: Characterization of disease-specific factors for inflammatory bowel disease

Disease-specific factors	Cases	Controls	*p*
Subjects. n	623	487	-
Calprotectin. µg/g stool	45.31 [15.22. 145.07]	19.2 [9. 42.62]	$< 10^{-5}$
Bristol stool scale. 1-7	4 [4. 4]	4 [4.4]	$< 10^{-5}$

Data is displayed as median [Interquartile Range]. Controls were recruited as first degree relatives from cases without IBD. Statistical significance was evaluated using Wilcoxon-test. Abbreviations: IBD. Inflammatory bowel disease

Beta diversity analysis for type 2 diabetes and inflammatory bowel disease

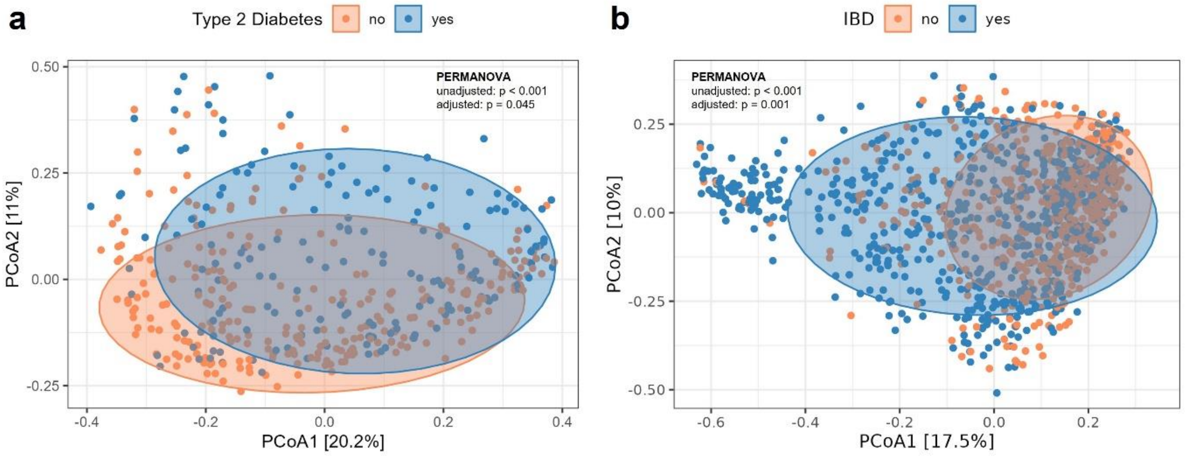

Figure S2: PCoA plots of case-control microbial beta diversity analysis for T2D and IBD.

This figure displays PCoA plots corresponding to microbial beta diversity analysis using the Bray Curtis dissimilarity approach between (a) Type 2 diabetes and (b) inflammatory bowel disease and their respective control groups. Statistical significance of differences was tested using a permutational multivariate analysis of variance (PERMANOVA) both unadjusted and adjusted for age, sex and BMI class with p-values provided within the PCoA plots. Abbreviation: IBD = inflammatory bowel disease.

Addition to the association between biomedical and lifestyle factors and age-at-disease-onset

Table S6A: Hazard ratios from Cox-regression models of biomedical and lifestyle factors in relation to age-at-T2D-onset

Biomedical and lifestyle factors	Comparison	Thresholds	Hazard Ratio [95% CI]
HEI-EPIC	Moderate	(44. 52]	0.64 [0.396-1.034]
	High	(52. 81]	0.453 [0.284-0.722]
MDS	Moderate	(4. 5]	0.947 [0.669-1.340]
	High	(5. 9]	1.495 [1.025-2.180]
Triglycerides	Moderate	(74. 118]	1.166 [0.579-2.349]
	High	(118. 988]	1.249 [0.647-2.412]
Smoker	Smoker		1.819 [1.248-2.651]

Thresholds of reference levels. which is the lowest level for all lifestyle factors: HEI-EPIC: [19. 44]; MDS: [1. 3]; Triglycerides: [28. 74].

Table S6B: Hazard ratios from Cox-regression models of biomedical and lifestyle factors in relation to age-at-IBD-onset

Biomedical and lifestyle factors	Level	Thresholds	Hazard Ratio [95% CI]
Scaled energy intake	Moderate	(28.5. 38.2]	0.997 [0.82-1.213]
	High	(38.2. 208]	1.023 [0.823-1.271]
Total energy intake	Moderate	(1.98e+03. 2.6e+03]	0.838 [0.692-1.016]
	High	(2.6e+03. 1.08e+04]	0.815 [0.652-1.019]
HEI-EPIC	Moderate	(44. 52]	0.994 [0.823-1.199]
	High	(52. 80]	0.807 [0.663-0.982]
MDS	Moderate	(4. 5]	0.932 [0.772-1.125]
	High	(5. 9]	0.691 [0.567-0.841]
Alcohol	Moderate	(1.74. 9.09]	0.727 [0.604-0.876]
	High	(9.09. 264]	0.488 [0.397-0.599]
Smoker	Smoker (UC)		0.467 [0.277-0.785]
	Smoker (CD)		0.836 [0.604-1.158]
Shannon index	Moderate	(2.07. 2.33]	0.638 [0.527-0.771]
	High	(2.33. 3.11]	0.587 [0.485-0.711]
Chao1 index	Moderate	(40. 48]	0.612 [0.508-0.738]
	High	(48. 84]	0.525 [0.432-0.639]
Sports activity	Moderate	(2.15. 5.03]	0.974 [0.805-1.179]
	High	(5.03. 63.5]	1.016 [0.836-1.234]
TV watching	Moderate	(5.5. 12]	0.951 [0.786-1.151]
	High	(12. 60]	0.848 [0.697-1.032]
Sleep	Moderate	(7.25. 8]	0.934 [0.772-1.13]
	High	(8. 29]	1.208 [0.992-1.471]
Bristol stool scale	2	mild constipation	1.374 [0.734-2.573]
	3	severe constipation	1.002 [0.373-2.691]
	4	missing fiber	1.849 [1.315-2.601]
	5	mild diarrhea	2.096 [1.61-2.728]
	6	severe diarrhea	2.922 [1.976-4.321]
Calprotectin	Moderate	(15.8. 57.1]	1.204 [0.976-1.485]
	High	(57.1. 676]	2.213 [1.819-2.691]
Everyday activity	Moderate	(8.9. 17]	1.048 [0.861-1.276]
	High	(17. 108]	0.833 [0.679-1.022]

Thresholds of reference levels. which is the lowest level for all lifestyle factors: Total energy intake: [671. 1.98e+03]; Scaled energy intake: [11.2. 28.5]; HEI-EPIC: [22. 44]; MDS: [0. 4]; Alcohol: [11.2. 28.5]; Shannon index: [0. 2.07]; Chao1 index: [1. 40]; Sports activity: [0. 2.15]; TV watching: [0. 5.5]; Sleep: [0. 7.25]; Calprotectin: [9. 15.8]; Everyday activity: [0. 8.9].

References

1 Sharma A. Junge O. Szymczak S. et al. Network-based quantitative trait linkage analysis of microbiome composition in inflammatory bowel disease families. *Front Genet* 2023; **14:** 1048312. https://doi.org/10.3389/fgene.2023.1048312.

2 Wang J. Thingholm LB. Skiecevičienė J. et al. Genome-wide association analysis identifies variation in vitamin D receptor and other host factors influencing the gut microbiota. *Nat Genet* 2016; **48:** 1396–406. https://doi.org/10.1093/hmg/ddw134.

3 Demetrowitsch TJ. Petersen B. Keppler JK. et al. Validation of a two-step quality control approach for a large-scale human urine metabolomic study conducted in seven experimental batches with LC/QTOF-MS. *Bioanalysis* 2015; **7:** 103–12. https://doi.org/10.4155/bio.14.270.

4 Seoudy AK. Schlicht K. Kulle A. et al. A PROSPECTIVE ANALYSIS OF THE METYRAPONE SHORT TEST USING TARGETED AND UNTARGETED METABOLOMICS. *Neuroendocrinology* 2023. https://doi.org/10.1159/000529146.

5 Kind T. Fiehn O. Seven Golden Rules for heuristic filtering of molecular formulas obtained by accurate mass spectrometry. *BMC Bioinformatics* 2007; **8:** 105. https://doi.org/10.1186/1471-2105-8-105.

6 Wishart DS. Guo A. Oler E. et al. HMDB 5.0. The Human Metabolome Database for 2022. *Nucleic Acids Res* 2022; **50:** D622-D631. https://doi.org/10.1093/nar/gkab1062.

7 Luan H. Ji F. Chen Y. Cai Z. statTarget. A streamlined tool for signal drift correction and interpretations of quantitative mass spectrometry-based omics data. *Anal Chim Acta* 2018; **1036:** 66–72. https://doi.org/10.1016/j.aca.2018.08.002.

8 Andrees V. Wolf S. Augustin M. Mohr N. Augustin J. Regional variations and prevalence of psoriasis in Germany from 2010 to 2017. A cross-sectional. spatio-epidemiological study on ambulatory claims data. *BMJ Open* 2021; **11:** e047806. https://doi.org/10.1136/bmjopen-2020-047806.

9 World Health Organization. Obesity. Preventing and managing the global epidemic. Report of a WHO consultation. *World Health Organ Tech Rep Ser* 2000; **894:** i-xii. 1-253.

10 Mallick H. Rahnavard A. McIver LJ. et al. Multivariable association discovery in population-scale meta-omics studies. *PLoS Comput Biol* 2021; **17:** e1009442. https://doi.org/10.1371/journal.pcbi.1009442.

11 Groeneveld M. Borgmann D. Brückner A. Wüstefeld-Würfel M. Acker I. [The food pyramid]. [Compendium for nutrition professionals]. Bonn: German Federal Agency for Agriculture and Food. 2020.

12 Rüsten A von. Boeing H. Flothkötter M. [The evaluation of food intake using a "Healthy Eating Index"]. German: Die Bewertung der Lebensmittelaufnahme mittels eine "Healthy Eating Index". *Ernährungsumschau* 2009: 450–56.

13 Trichopoulou A. Costacou T. Bamia C. Trichopoulos D. Adherence to a Mediterranean diet and survival in a Greek population. *N Engl J Med* 2003; **348:** 2599–608. https://doi.org/10.1056/NEJMoa025039.

14 Tosti V. Bertozzi B. Fontana L. Health Benefits of the Mediterranean Diet. Metabolic and Molecular Mechanisms. *J Gerontol A Biol Sci Med Sci* 2018; **73:** 318–26. https://doi.org/10.1093/gerona/glx227.

15 Trichopoulou A. Orfanos P. Norat T. et al. Modified Mediterranean diet and survival. EPIC-elderly prospective cohort study. *BMJ* 2005; **330:** 991. https://doi.org/10.1136/bmj.38415.644155.8F.
